# High‐Efficiency Water Collection of Superhydrophobic Condensation Absorber

**DOI:** 10.1002/advs.202417024

**Published:** 2025-02-10

**Authors:** Defeng Yan, Junyi Lin, Yang Chen, Xiaolong Yang, Yao Lu, Jinlong Song

**Affiliations:** ^1^ State Key Laboratory of High‐performance Precision Manufacturing Dalian University of Technology Dalian 116024 China; ^2^ Key Laboratory for Micro/Nano Technology and System of Liaoning Province Dalian University of Technology Dalian 116024 China; ^3^ College of Mechanical and Electrical Engineering Nanjing University of Aeronautics and Astronautics Nanjing 210016 China; ^4^ Department of Chemistry School of Physical and Chemical Sciences Queen Mary University of London London E1 4NS UK

**Keywords:** atmospheric water collection, high efficiency, high‐frequency surface refreshing, superhydrophobic surface

## Abstract

The atmosphere contains ≈1.3 billion tons vapor that can be condensed to obtain water, which has the promise of alleviating the water crisis. However, condensed droplets are difficult to shed from the condensation surface that means a low surface refreshing frequency, showing the low water collection rate and efficiency. Here, this limitation is successfully overcome by proposing a novel superhydrophobic condensation absorber (SCA). All surfaces of the SCA are superhydrophobic but covered with a series of superhydrophilic through pores and superhydrophilic points which enabled the SCA with a rapid droplet nucleation capability. The whole condensation processes exhibit that the SCA has  the extremely small droplet shedding volume and the highly frequent surface refreshing, which are 0.00003 and 1.1× 10^6^ times that of the existing water collection method, respectively. The water collection rate of SCA is superior than that of the existing water collection methods, reaching to 80 mg cm^−2^ h^−1^ at the subcooling temperature of only 10 °C. In addition, the collected water by this SCA is clean without any contaminant. This high‐efficiency and eco‐friendly water collection method will maximize the acquisition of clean water from atmosphere, which has a strong implication for the people suffering from the freshwater crisis.

## Graphical abstract

The combination of the low nucleation barrier, small droplet shedding volume, and high‐frequency surface refreshing makes the superhydrophobic condensation absorber (SCA) has a high water collection rate. In addition, this atmospheric water collection method based on the droplet jetting phenomenon enables excellent water collection capacity on the top wall, side wall, and bottom wall of SCA.

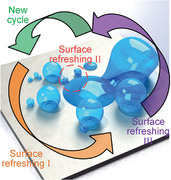

## 1 Introduction

As the rapid increasing of global population, the 193 member states of the United Nations adopted 17 Sustainable Development Goals (SDGs), where the Goal 6 of SDGs stated that water scarcity seriously restricted the human sustainable development.^[^ ^1^, ^2^ ^]^ The main reason that the existing methods for obtaining the clean water, such as inter‐basin water diversion, seawater desalination, and sewage purification, are difficult to popularize which results from the high‐cost and the high energy consumption.^[^ ^3^, ^4^, ^5^ ^]^ Therefore, people urgently need a low‐cost and energy‐saving method to obtain the clean water.

In nature, the atmosphere stores a large amount of sustainably regenerated water resources in the form of vapor or fog, which is ≈1.3 × 10^9^ tons, accounting for 10% of all other freshwater resources.^[^ ^6^, ^7^ ^]^ If these water vapor can be collected and utilized, it can alleviate the water crisis.^[^ ^8^ ^]^ The organism and phenomena in nature have inspired people to collect vapor from the atmosphere, e.g., the leaves of plant generate the dew on a damp night or morning. This means that when an object is located in a damp environment and its surface temperature is lower than the dew temperature, the vapor in the atmosphere will condense on the object surface and form the condensation water.^[^ ^9^ ^]^ Researchers found that the dropwise condensation usually exhibited a better water collection rate than the filmwise condensation. This was because the filmwise condensation form had a thicker insulation layer which led to a lower heat transfer performance and a lower surface refreshing frequency.^[^ ^10^, ^11^, ^12^ ^]^ However, even though the condensation surface is dropwise condensation, its surface refreshing frequency is still not high enough because the condensed droplets need to grow to a certain size with ≈2.7 mm to overcome the capillary force and shed from the condensation surface by the gravity.^[^ ^13^, ^14^ ^]^ To increase the surface refreshing frequency, the micro‐/nano‐structured superhydrophobic surface with the droplet jumping phenomenon and the slippery liquid‐infused porous surface (SLIPS) were proposed.^[^ ^15^, ^16^, ^17^, ^18^, ^19^ ^]^ Nevertheless, the landing position of the shedding droplets on the micro‐/nano‐structured superhydrophobic surface was random, resulting in a significant loss of the shedding droplets.^[^ ^20^ ^]^ In addition, the shedding droplets would take away a lot of lubricant on the SLIPS, which caused a contamination of the collected water.^[^ ^21^, ^22^ ^]^ Therefore, if we can develop a novel water collection method with high‐frequency surface refreshing and high‐efficiency collection of the shedding droplet but without any contamination will be very meaningful to the world, especially for the people suffering from the freshwater crisis.

In this work, we designed a novel superhydrophobic condensation absorber (SCA) which was filled with water and inspired by the quick shedding phenomenon of water droplets from the lotus leaf surface. All surfaces of the SCA were superhydrophobicity but covered with a series of superhydrophilic through pores and superhydrophilic points. Then, the dynamic behavior of the condensed droplets on the SCA and their influencing factors including the relative ambient humidity and surface temperature were investigated by an environmental scanning electron microscope and a camera, respectively. The condensed droplets could quickly and frequently shed from the SCA, greatly increasing its surface refreshing frequency, which made it possible to collect a large amount of water from the atmosphere even under a lower subcooling. More interesting is that this high‐efficiency and environment‐friendly water collection method can generate the abundant freshwater resources and promote the human sustainable development.

## 2 Results and Discussion

### 2.1 Ultra‐Rapid Droplet Jetting Enabled High‐Frequency Surface Refreshing

**Figure 1:**
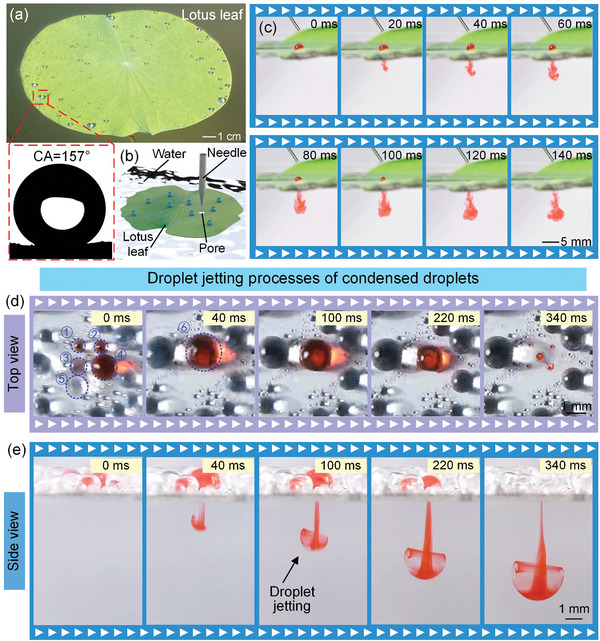
Droplet jetting phenomena on the lotus leaf and a superhydrophobic Al sheet with superhydrophilic pore. a) A lotus leaf in the pond. b) Schematic of fabricating through pore on the lotus leaf using a needle. c) When a dyed 15 µL droplet contacted the pore on the lotus leaf, it was quickly absorbed into this pore and generated a droplet jetting phenomenon. The entire droplet jetting process was ≈140 ms. d) Top view of the condensed droplets on a superhydrophobic Al sheet with superhydrophilic through pore. e) Side view of the condensed droplets on a superhydrophobic Al sheet with superhydrophilic through pore. The diameters of superhydrophilic through pore and superhydrophilic point were 300 µm and 100 µm, respectively. The ambient temperature, relative ambient humidity, and condensation surface temperature for this experiment were 26 °C ± 1 °C, 70% ± 2.5%, and 10 °C ± 1 °C, respectively.

Lotus leaf is a common superhydrophobic surface in nature, where a water droplet has a contact angle greater than 150 ° and is easily shed from this surface (**Figure** **1**a).^[^ ^23^ ^]^ When the lotus leaf was subjected to the insect infestation or mechanical damage, a through pore will form on its surface (Figure 1b; Figure S3, Supporting Information). Once a water droplet on the lotus leaf contacted this pore, it would be absorbed in the pore and entered the water in a form of droplet jetting within 140 ms, keeping the surface cleaning (Figure 1c; Video S1, Supporting Information). Inspired by this phenomenon, we guessed that if the condensed droplet on the surface could spontaneously shed from the condensation surface in the form of this ultra‐rapid droplet jetting, it will facilitate the shedding of condensed droplets. Therefore, we first investigated whether a superhydrophobic surface with superhydrophilic pore could generate the condensed droplets and these condensed droplets whether could be easily and rapidly shed from the condensation surface by superhydrophilic pore in the form of droplet jetting. To verify this hypothesis, we made a condensation platform and fabricated a superhydrophobic Al sheet with a superhydrophilic through pore with 300 µm diameter via the combination of the laser processing and the FAS modification (Figures S4 and S5, Supporting Information).^[^ ^24^, ^25^, ^26^ ^]^ In order to facilitate the observation of the droplet jetting, we fabricated four superhydrophilic points with 100 µm diameter around superhydrophilic through pore via the laser and placed a 1.5 µL dye droplet on one of these superhydrophilic points. After 30 min condensation, the condensed droplets could be clearly observed on superhydrophobic surface and four superhydrophilic points. These droplets gradually grew and coalesced with the surrounding other condensed droplets with the increasing time (Figures S6 and S7, Supporting Information). After 90 min, the droplet ⑤ on superhydrophobic surface coalesced with the droplet ③ on superhydrophilic point. During the coalescence process, the coalesced droplets generated the droplet oscillation because of the surface energy releasing, which caused the coalesced droplet contacted the droplet ①, droplet ②, and droplet ④ on superhydrophilic points and ultimately coalesced to form a large droplet ⑥ (Figure S8, Supporting Information). Once this droplet ⑥ contacted superhydrophilic pore, it was easily and rapidly entered the water through the pore in the form of droplet jetting within 340 ms, as shown in Figure 1d,e, and Video S2 (Supporting Information). Therefore, superhydrophobic surface with superhydrophilic pore could generate the condensed droplets. The generated condensed droplets can easily and rapidly shed from the condensation surface by superhydrophilic pore in the form of droplet jetting, which has the potential to be used for collecting water from the atmosphere.

**Figure 2:**
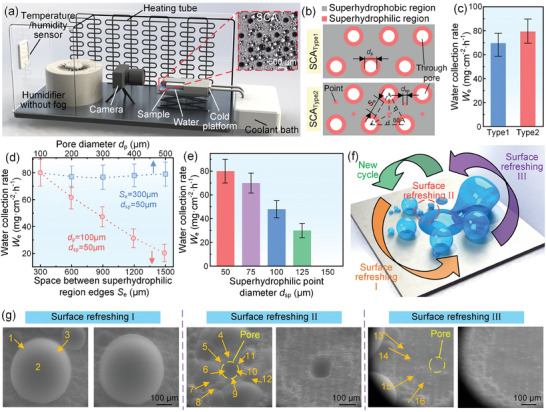
The SCA had the high‐frequency surface refreshing which resulted from the ultra‐rapid droplet jetting. a) A closed chamber was used to observe condensation processes on the SCA and conduct water collection experiments. b) Schematic of the SCA_type1_ without superhydrophilic points and the SCA_type2_ with superhydrophilic points. Superhydrophilic point diameter *d*_sp_ of 50 µm. c) The water collection rate of the SCA_type1_ and the SCA_type2_. d) The variation of water collection rate with the pore diameter and the space between superhydrophilic region edges. e) The variation of water collection rate with superhydrophilic point diameter. In these water collection experiments, the ambient temperature, relative ambient humidity, and sample surface temperature were 26 °C ± 1 °C, 70% ± 2.5%, and 10 °C ± 1 °C, respectively. f) Schematic of three surface refreshing types on the SCA. g) ESEM images of three surface refreshing types on the SCA.

We then established a condensation experiment system to investigate the condensation performance of our proposed method that inspired by the ultra‐rapid droplet jetting phenomenon, as shown in **Figure** **2**a. The SCA was the core part of the condensation experiment system Figure S9 (Supporting Information). In the droplet jetting experiment shown in Figure 1e, we found that the droplet nucleation rate on superhydrophilic point was much faster than that on superhydrophobic surface. Therefore, we proposed that if superhydrophilic points were fabricated at the incenter of triangle of superhydrophilic staggered arrangement pore array, it will be beneficial to increase the water collection rate because of the increased droplet nucleation rate during the condensation processes (Figure 2b). The influence of the SCA_type1_ without superhydrophilic points and the SCA_type2_ with superhydrophilic points on the water collection rate was studied. The results showed that the water collection rates of SCA_type1_ and SCA_type2_ were 69.5 mg cm^−2^ h^−1^ and 80.0 mg cm^−2^ h^−1^, respectively, for the same pore diameter *d*_p_ of 300 µm and the same space *S*_e_ of 300 µm between superhydrophilic region edges, as shown in Figure 2c. It indicated that the superhydrophilic points indeed could increase the water collection rate. Then, the SCA_type2_ was used as the SCA in the following work.

We further investigated the influence of the SCA structure parameters on the water collection rate and found that the increasing *S*_e_ made the condensed droplets difficult to be captured by superhydrophilic pores, resulting in the loss of the shedding droplet. Differently, the *d*_p_ from 100 µm to 500 µm had almost no effect on the water collection rate (Figure 2d; Figure S10, Supporting Information). This was because that the shedding size of the condensed droplets on the SCA was small and the shedding droplets could be completely absorbed by superhydrophilic pores with *d*_p_ of 100 µm‐500 µm. In addition, it can be seen that if the pore area was constant, the pore shape was also insignificant for the droplet jetting process and the water collection rate (Figures S11 and S12 and Video S3, Supporting Information). However, the diameter *d*_sp_ of superhydrophilic point had a significant influence on the water collection rate. The water collection rate decreased with the increasing *d*_sp_, which resulted from that the increasing *d*_sp_ would decrease the area of superhydrophobic region, as shown in Figure 2e. When the *d*_sp_ was larger than 150 µm, the condensed droplet on superhydrophilic point easily formed the liquid bridge with the water on superhydrophilic area around superhydrophilic pore, resulting in the loss of water collection capability. Therefore, we chose the *d*_p_ of 100 µm, the *S*_e_ of 300 µm, and the *d*_sp_ of 50 µm as the structure parameters of the SCA (Figure S13, Supporting Information).

We then carefully observed the condensation processes on the SCA by an environmental scanning electron microscope (ESEM) and found that the surface refreshing of the SCA was very frequent and could be summarized into three types, as shown in Figure 2f,g, Figure S14 and Videos S4–S6 (Supporting Information). For the surface refreshing I, once the condensed droplets on superhydrophobic surface contacted the condensed droplet on superhydrophilic point, the condensed droplets on superhydrophobic surface would rapidly coalesce with the condensed droplet on superhydrophilic point under the Laplace force, refreshing the SCA surface. For the surface refreshing II, once the condensed droplets around superhydrophilic pore contacted superhydrophilic region around superhydrophilic pore, they would be quickly absorbed into superhydrophilic pore, refreshing the SCA surface. For the surface refreshing III, once grown condensed droplet on superhydrophilic point contacted superhydrophilic region around superhydrophilic pore, it would rapidly shed from the SCA surface and be absorbed into superhydrophilic pore, refreshing the SCA surface. In addition, we also calculated the maximum droplet shedding sizes of three surface refreshing types, which could be expressed as,

(1) $$D_{\max-I}=D_{\max-\mathrm{II}}=\frac{{\left(S_{e}+d_{r}\right)}^{2}+3{\left(S_{e}\right)}^{2}-3{\left(d_{\mathrm{spr}}\right)}^{2}}{6\left(S_{e}+d_{\mathrm{spr}}\right)}$$

(2) $$D_{\max-\mathrm{III}}=\frac{\left(2\sqrt{3}-3\right)d_{r}+2\sqrt{3}S_{e}}{3}$$

where *D*_max‐I_, *D*_max‐II_, and *D*_max‐III_ are the maximum droplet shedding sizes of the surface refreshing I, surface refreshing II, and surface refreshing III, respectively (Figure S15, Supporting Information). *d*_r_ and *d*_spr_ are the diameter of superhydrophilic region around superhydrophilic pore and the diameter of superhydrophilic region around superhydrophilic point, which are 300 µm and 150 µm, respectively. Combined with Equations (1) and (2), the *D*_max‐I_, *D*_max‐II_, and *D*_max‐III_ could be calculated by MATLAB, which were 208 µm, 208 µm, and 393 µm, respectively. Therefore, the extremely small droplet shedding size resulted in the high‐frequency droplet shedding and high‐frequency surface refreshing, which meant that this method could timely phase‐separate by separating the condensed droplets, vapor, and condensation surface.^[^ ^15^, ^27^ ^]^

### 2.2 Dynamic Analysis of Condensed Droplets on the SCA

**Figure 3:**
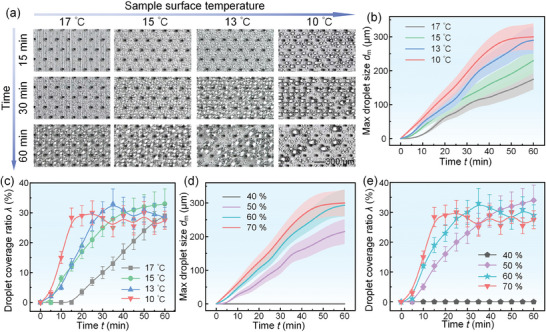
Dynamic behaviors of condensed droplets on the SCA. a) The condensation processes on the SCA at different sample surface temperatures, where the ambient temperature and the relative ambient humidity were 26 °C ± 1 °C and 70% ± 2.5%. b) The variation of the maximum droplet size on the SCA with the time at different sample surface temperatures. c) The variation of the droplet coverage ratio on the SCA with the time at different sample surface temperatures. d) The variation of the maximum droplet size on the SCA with the time at different relative ambient humidities. e) The variation of the droplet coverage ratio on the SCA with the time at different relative ambient humidities. In the condensation experiments with different relative ambient humidities, the ambient temperature and the sample surface temperature were 26 °C ± 1 °C and 10 °C ± 1 °C.

To investigate the influence of different conditions on the condensation performance of SCA, we then studied the dynamic behaviors of the condensed droplets on the SCA under the different relative ambient humidities and sample surface temperatures, respectively. We first adjusted the temperature of the cold plate to achieve different sample surface temperatures (Table S1 and Video S7, Supporting Information). Under the different sample surface temperatures, the dynamic behaviors of the condensed droplets were different, as shown in **Figure** **3**a. When the sample surface temperature was 17 °C, the surface subcooling temperature was only ≈3 °C. The condensed droplets could be observed on the SCA surface after ≈30 min. However, the condensed droplets on the SCA surface could be observed about only 15 min at the 15 °C sample surface temperature. The time to observe the condensed droplets was decreased with the decreasing sample surface temperature. In addition, we found that when the sample surface temperature was 10 °C, the condensed droplets on superhydrophilic point would quickly shed from the SCA surface and enter the pore after 30 min, leaving a refreshed surrounding region around this pore (Figure 3a). We chose a 2 mm × 2 mm area as the observation region to carefully observe and measure the maximum size of the condensed droplets on the SCA with the different sample surface temperatures. The results indicated that the maximum droplet size was less than 350 µm, which was consistent with the theoretical calculation value in the section 3.1, as shown in Figure 3b. We also calculated the droplet coverage ratio which was the ratio of the area of the condensed droplets to the total area of SCA in the observation region. It was exciting that the droplet coverage ratio on the SCA was always less than 35% at the different sample temperatures (Figure 3c; Figure S16, Supporting Information). Moreover, we also studied and observed the dynamic behaviors of the condensed droplets on the SCA at different relative ambient humidities, where the ambient temperature and the sample temperature were 26 °C ± 1 °C and 10 °C ± 1 °C (Figure S17, Supporting Information). When the relative ambient humidity was higher than 40%, the condensed droplets could be observed within 60 min with the maximum droplet size less than 350 µm and the droplet coverage ratio less than 35% (Figure 3d,e; Figure S18 and Video S8, Supporting Information). However, when the relative ambient humidity was 40%, there were no condensed droplets observed on the SCA within 60 min due to the too low subcooling temperature (Table S2, Supporting Information).

**Figure 4:**
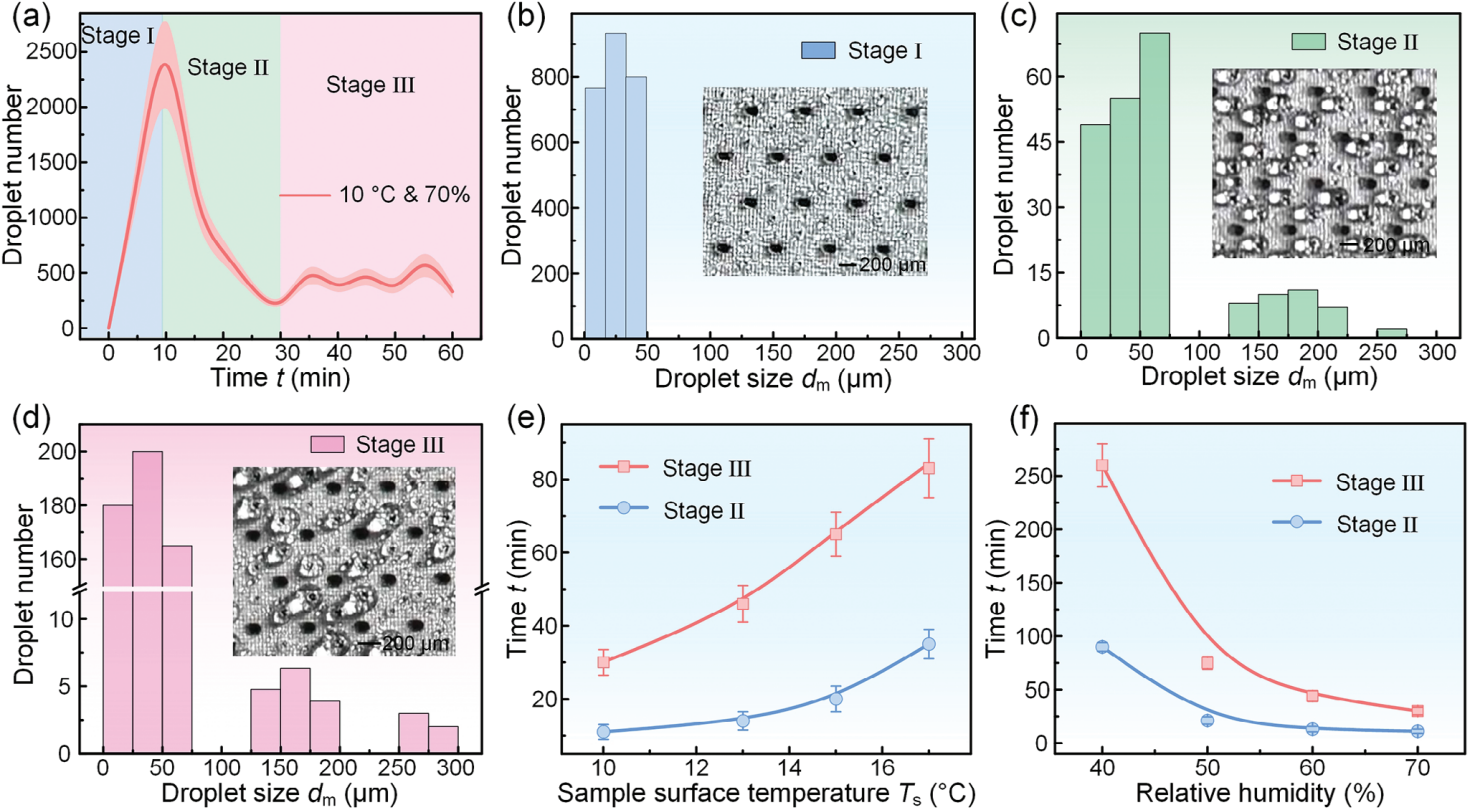
The three stages of condensation processes on the SCA. a) The variation of the droplet number on the SCA with the time within a 2 mm × 2 mm area. According to the droplet size and the droplet number, the condensation process was divided into three stages: condensed droplet nucleation stage (Stage I), condensed droplet growth stage (Stage II), and condensed droplet absorption stage (Stage III). The ambient temperature, relative ambient humidity, and sample surface temperature were 26 °C ± 1 °C, 70% ± 2.5%, and 10 °C ± 1 °C, respectively. b) The droplet size and the droplet number on the SCA during the Stage I. c) The droplet size and the droplet number on the SCA during the Stage II. d) The droplet size and the droplet number on the SCA during the Stage III. e) The variation of the time to reach the Stage I and Stage II with the increasing sample surface temperature, where the ambient temperature and the relative ambient humidity were 26 °C ± 1 °C and 70% ± 2.5%. f) The variation of the time to reach the Stage I and Stage II with the increasing relative ambient humidity, where the ambient temperature and the sample surface temperature were 26 °C ± 1 °C and 10 °C ± 1 °C.

According to the dynamic analysis of the condensed droplets, we divided the condensation processes of the SCA into three stages: condensed droplet nucleation stage (Stage I), condensed droplet growth stage (Stage II), and condensed droplet absorption stage (Stage III). We chose the experiment condition of the ambient temperature of 26 °C, relative ambient humidity of 70%, and sample temperature of 10 °C to further analyze the dynamic processes of the condensed droplets on the SCA, as shown in **Figure** **4**a. In the Stage I, since the nucleation barrier was much lower on superhydrophilic point than on superhydrophobic region, the condensed droplets first nucleated and were observed on superhydrophilic points. During this process, the condensed droplet size was very small less than 50 µm, but the condensed droplet number was large and rapidly reached 2000 within 10 min (Figure 4b). In addition, there was almost no visible surface refreshing in the stage I. After 10 min, the condensation process reached the Stage II. Once the gradually growth droplets on superhydrophobic region contacted the droplet on superhydrophilic point, they would quickly coalesce with the droplet on superhydrophilic point, refreshing the region around this point. During this stage, we would observe the significant surface refreshing phenomena in the form of surface refreshing I, which caused that the droplet number and the droplet size significantly decreased and increased, respectively (Figure 4c). As the droplets on superhydrophilic point gradually grew, they would contact superhydrophilic pores, which reached the Stage III. Once these droplets contacted superhydrophilic pores, they would be quickly absorbed into the SCA, refreshing the region around the pores and starting a new condensation cycle, as shown in Figure 4d. During this stage, the phenomena of the surface refreshing II and surface refreshing III could be frequently observed on SCA. In addition, we also investigated the variations of the droplet number on the SCA with the condensation time at different sample surface temperatures and different relative ambient humidities (Figure S19, Supporting Information). The results showed that the time to reach the Stage II and Stage III increased from 11 min to 35 min and from 30 min to 83 min with the sample surface temperature increased from 10 to 17 °C, and decreased from 90 min to 11 min and from 260 min to 30 min with the relative ambient humidity increased from 40% to 70%, as shown in Figure 4e,f. Therefore, it can be seen that the difference between the sample surface temperature and ambient temperature and the relative ambient humidity are important factors that affect the condensation processes of the SCA. When the SCA was placed in a humid environment, it can quickly generate condensed droplets on the surface and frequently shed these condensed droplets from the surface in the form of the droplet jetting, which is beneficial for improving the water collection rate.^[^ ^27^ ^]^

### 2.3 High‐Efficiency Water Collection

**Figure 5:**
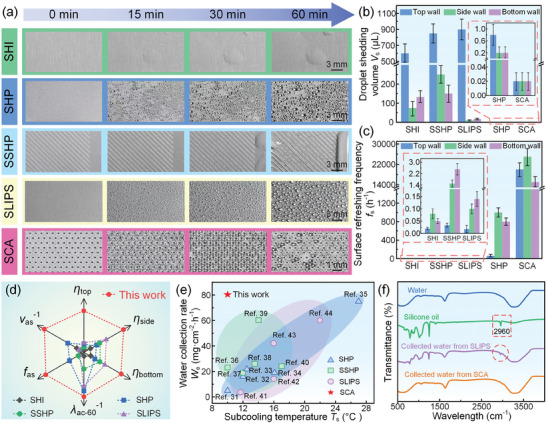
The SCA had a high‐efficiency water collection capability. a) The water collection processes of the SHI, SHP, SSHP, SLIPS, and SCA at the top wall. In these water collection experiments, the ambient temperature, relative ambient humidity, and sample surface temperature were 26 °C ± 1 °C, 70% ± 2.5%, and 10 °C ± 1 °C, respectively. b) Droplet shedding size of the different samples on the top wall/side wall/bottom wall. c) Surface refreshing frequency of the different samples on the top wall/side wall/bottom wall. d) Performance comparison of the water collection efficiency of the top wall/ side wall/ bottom wall, the average droplet shedding volume, the average surface refreshing frequency, and the average droplet coverage ratio after 60 min condensation of these five surfaces. e) Water collection rate of the different water collection methods. The detailed data of these literatures is included in Table S8 (Supporting Information). f) FTIR spectrum of the clean water, the silicone oil, the collected water from the SLIPS, the collected water from the SCA.

Previous researches showed that superhydrophilic surface (SHI), superhydrophobic surface (SHP), superhydrophobic/superhydrophilic hybrid pattern surface (SSHP), and SLIPS had a good water collection capability.^[^ ^28^, ^29^, ^30^, ^31^, ^32^, ^33^, ^34^, ^35^, ^36^, ^37^, ^38^, ^39^, ^40^, ^41^, ^42^, ^43^, ^44^, ^45^, ^46^ ^]^ Almost all researches placed the water collection surface perpendicular to the horizontal plane, relying on gravity to make the condensed droplets shed. However, in the practical application, an object is usually composed of the top wall, side wall, and bottom wall. If only the side wall is considered to collect water which means the top wall and side wall are ignored, seriously reducing the water collection capability. We fabricated four surfaces as the control group and conducted the water collection experiments of the SHI, SHP, SSHP, SLIPS, and SCA on the top wall, side wall, and bottom wall (**Figure** **5**a; Figures S20–S23 and Videos S9–S11, Supporting Information).

In these five surfaces, the condensed droplets were the most difficult to shed from the SHI but easily shed from the SHP and the SCA because of different surface wettability. By measuring the shedding droplet volume, we found that the droplet shedding volume of the SCA on the top wall/side wall/bottom wall was only 0.03 µL/0.03 µL/0.03 µL, which was 0.00005/0.0004/0.00023, 0.0375/0.15/0.15, 0.00004/0.00012/0.0002, and 0.00003/0.003/0.00177 times of that of SHI, SHP, SSHP, and SLIPS, (Figure 5b; Figure S24, Supporting Information). We then counted the surface refreshing frequency of these five surfaces and found that the surface refreshing frequency of this SCA was as high as 20000 h^−1^/25000 h^−1^/15000 h^−1^ on the top wall/side wall/bottom wall, which was 1 ×  10^6^/3.1 × 10^5^/3 × 10^5^, 333/25/19, 6 × 10^5^/1.7 × 10^4^/6000, and 1.1 × 10^6^/2.5 × 10^5^/1.1 × 10^5^ times of that of SHI, SHP, SSHP, and SLIPS (Figure 5c). The high surface refreshing frequency of the SCA was due to the extremely small droplet shedding size and rapidly phase‐separation form. In addition, we also calculated the droplet coverage ratio of these five surfaces at the top wall/side wall/bottom wall and found that the droplet coverage ratio of the SCA was always less than 35% that originated from the high‐frequency droplet shedding and high‐frequency surface refreshing, which would decrease the influence of thermal resistance generated by the condensed droplets on the surface, as shown in Figure S25 (Supporting Information). Although the SHP also exhibited a high surface refreshing frequency and a low droplet coverage ratio, since the droplet shedding form of the SHP was the droplet jumping that originated from the surface energy releasing, resulting in a significant loss of the shedding droplets. To objectively evaluate the water collection capability of these different surfaces, we developed a performance figure of merit for this condensation experiment system, as shown in Figure S26 and Tables S3–S7 (Supporting Information). The water collection efficiency *η* was used to appraise the water collection capability from the atmosphere, which could be described as follows,

(3) $$\eta =\frac{W_{e}}{W_{t}}\times 100\%$$

where *W*_e_ and *W*_t_ are the experimental water collection rate and the theoretical water collection rate of the sample surface.^[^ ^34^ ^]^ The experimental water collection rate was calculated as follows:

(4) $$W_{e}=\frac{m_{e}}{A_{e}\times t_{e}}$$

where *m*_e_, *A*_e_, and *t*_e_ are the water collection mass, the collection area, and the collection time.^[^ ^7^ ^]^ The theoretical water collection rate of the sample surface can be expressed as follows:

(5) $$W_{t}=W_{m,\mathrm{air}}\times \Delta k_{\mathrm{vapor}}$$

where *W*_m, air_ and Δ*k*_vapor_ were the mass transfer coefficient of air and the difference in the mass fraction of vapor and air between the air and the sample surface.^[^ ^34^ ^]^ The model and detailed calculation process are shown in Figure S26 (Supporting Information). After calculation, we comprehensively compared the water collection efficiency of the top wall (*η*_top_)/ side wall (*η*_side_)/ bottom wall (*η*_bottom_), the average droplet shedding volume *V*_as_, the average surface refreshing frequency *f*_as_, and the average ratio *λ*_ac‐60_ of droplet coverage after 60 min condensation of these five surfaces. It could be seen that these performances of the SCA were superior than that of the other water collection methods (Figure 5d). Moreover, we also calculated the subcooling temperature and water collection rate on the SHP, SSHP, and SLIPS used in the reported literatures and compared their water collection performance with that of the SCA.^[^ ^32^, ^33^, ^34^, ^35^, ^36^, ^37^, ^38^, ^39^, ^40^, ^41^, ^42^, ^43^, ^44^, ^45^ ^]^ As everyone knows, a larger subcooling temperature can increase the water collection rate. The SCA had a water collection rate of 80 mg cm^−2^ h^−1^ at a subcooling temperature of 10 °C, while the SHP required the subcooling temperatures of 27 °C to achieve the almost equiv. water collection rate (Figure 5e). In addition, we found that the collected water from the SLIPS was seriously contaminated by the lubricant oil (Figure 5f; Figures S27 and S28 and Table S9, Supporting Information). However, the collected water from the SCA was very clean and could be directly used for actual production or daily life (Table S10, Supporting Information). In this work, we focused on the atmospheric water collection to show the timely phase‐separation of the condensed droplets, vapor, and condensation surface. In the southern China, the high‐temperature and high‐humidity environments are an ideal atmospheric water collection environment. Therefore, we believe that this fundamental research will be conducive to human efficiently and environmentally obtain freshwater resources in the future.^[^ ^46^ ^]^

## 3 Conclusion

In summary, we proposed a novel SCA based on a droplet jetting phenomenon which was inspired by the quick shedding of water droplets from the lotus leaf surface. All surfaces of the SCA were superhydrophobic but covered with a series of superhydrophilic through pores and superhydrophilic points. The dynamic behaviors of the condensed droplets on the SCA showed that a higher relative ambient humidity and a larger subcooling temperature were beneficial to improve the droplet shedding frequency. The whole condensation processes of the SCA could be divided into three stages which were the condensed droplet nucleation stage, condensed droplet growth stage, and condensed droplet absorption stage. In these three stages, the condensed droplet size and the droplet coverage ratio on the SCA were always less than 350 µm and 35%. We compared the water collection performance of the SCA with the existing other water collection methods and found that the SCA had the superior water collection rate of 80 mg cm^−2^ h^−1^ at the subcooling temperatures of 10 °C. The superior water collection rate was due to the high‐frequency droplet shedding and high‐frequency surface refreshing, which resulted from the low droplet coverage ratio and the small droplet shedding size. In addition, the collected water by the SCA was clean. Since the water collection mass can be easily improved by increasing the size of the SCA, this water collection method based on the ultra‐rapid droplet jetting phenomenon is very meaningful to the world.

## 4 Experimental Section

### Materials

Aluminum (Al, purity of 99.6%) sheets with 100 mm ×100 mm ×0.3 mm were purchased from Suzhou Hongfa Co., Ltd (China). Anhydrous ethanol, water‐soluble red dye, and anhydrous acetone were bought from Tianjin Kemio Chemical Reagent Co., Ltd (China). Silicone oil (10 cst viscosity at 25 °C) and Fluoroalkylsilane [FAS, C_8_F_13_H_4_Si(OCH_2_CH_3_)_3_] were provided from Degussa Co. Ltd (Germany). Polymethyl methacrylate (PMMA) boards were bought from Shangpin Acrylic Production Co., Ltd (China). Needle and acrylic plates were bought from the local market.

### Fabrication of Superhydrophobic Condensation Absorber (SCA) Based on Droplet Jetting Phenomenon

The fabrication processes of the SCA are shown in Figure S1 (Supporting Information). The Al sheet was cleaned by ultrasonic cleaner (F‐031SD, Shenzhen Fuyang Technology Co., Ltd., China) with acetone for 5 min to remove contaminant. Then, the both sides of the Al sheet were textured via a nanosecond laser (SK‐CX30, Shanghai Sanke Laser Technology Co. Ltd., China) to fabricate the rough structures. Next, the Al sheet after laser texturing was immersed in 2 wt.% FAS ethanol solution for 60 min and dried at 85 °C for 10 min to obtain superhydrophobic Al sheet with a 162° ± 2° water contact angle. Then, this nanosecond laser was used to drill superhydrophilic through pores on superhydrophobic Al sheet. Finally, a superhydrophobic Al sheet with a series of superhydrophilic through pores was folded into a box. After filling with water, a SCA_type1_ was obtained (Figure S1a, Supporting Information). In addition, superhydrophilic points were also fabricated at the incenter of triangle of superhydrophilic pore array by the laser and the corresponding folded box was named as SCA_type2_ (Figure S1b, Supporting Information). The fabrication processes of entire superhydrophilic surface (SHI), entire superhydrophobic surface (SHP), superhydrophilic/superhydrophobic hybrid pattern (SSHP), and SLIPS are shown in Figure S2 (Supporting Information).

### Sample Characterization

A scanning electron microscope (SEM, JSM‐6360LV, Japan) was used to observe the surface microstructure of the SCA. The element compositions of the SCA were characterized by an energy dispersive spectroscope (EDS, JSM‐6360LV, Japan). An environmental scanning electron microscope (ESEM, Prisma E, USA) was used to observe the condensation processes. Fourier transform infrared spectrum (FTIR, Nicolet iS50, U.S.A.) was used to characterize the chemical compositions of the collected water. An inductively coupled plasma emission spectrometer (Avio 220, Singapore) was used to characterize the Heavy metal ion concentration of the water. An UV‐Vis‐NIR Spectrophotometer (Solid Spec‐3700, Japan) was used to characterize the UV‐Vis‐NIR absorption spectra of samples. A total organic carbon analyzer (TOC‐L CPH, Japan) was used to analyze the total organic carbon content in water collected from the SCA and SLIPS. An optical contact angle meter (DSA100, Krüss, Germany) was used to measure the water contact angle (WCA) of samples in the air, which used a water droplet of 3 µL under ambient temperature. The droplet jetting processes were recorded through a camera (D7500, Nikon, Japan).

### Water Collection Experiments

The water collection experiments by dew were performed in a self‐made closed chamber which was composed of the PMMA boards at atmospheric pressure. The sample platform was placed on a vertically movable platform for easy observation of condensation phenomena on the different samples. An aluminum cold plate was inserted into the sample platform and contacted with the inner wall of the sample platform for better heat transfer. The temperature of the sample surface and cold plate was controlled by a circulation coolant bath with coolant (ethylene glycol, BOSCH). A thermocouple was fixed on the sample surface to measure the temperature of sample surface. The temperature of sample surface could vary from 5 to 21 °C. The chamber temperature and the chamber humidity were detected by a temperature/humidity sensor and were controlled by a humidifier without fog and a heating tube, respectively. In the water collection experiment, if not specified, the ambient temperature, the relative ambient humidity, and the sample surface temperature were 26 °C ± 1 °C, 70% ± 2.5%, and 10 °C ± 1 °C, respectively. All the water collection experiments were recorded by the camera (D7500, Nikon, Japan).

## Conflict of Interest

The authors declare no conflict of interest.

## Supporting information



Supporting Information

Supporting Information Video 1

Supporting Information Video 2

Supporting Information Video 3

Supporting Information Video 4

Supporting Information Video 5

Supporting Information Video 6

Supporting Information Video 7

Supporting Information Video 8

Supporting Information Video 9

Supporting Information Video 10

Supporting Information Video 11

## Data Availability

The data that support the findings of this study are available from the corresponding author upon reasonable request.
