# Supplementary material for: High‐Efficiency Water Collection of Superhydrophobic Condensation Absorber
Source: Adv Sci (Weinh). 2025 Feb 10;12(13):2417024. doi: 10.1002/advs.202417024 (PMC11967871; doi:10.1002/advs.202417024)
Supplement: Supplementary file 1 — Supporting Information [file ADVS-12-2417024-s003.docx]

Supporting Information

**High-efficiency water collection of superhydrophobic condensation absorber**

Defeng Yan^1, 2^, Junyi Lin^1^, Yang Chen^1^, Xiaolong Yang^3^, Yao Lu^4^, and Jinlong Song^1, 2*^

**Content**

**Figures**

Figure S1. Schematics of the fabrication processes of superhydrophobic condensation absorber (SCA) based on droplet jetting phenomenon.

Figure S2. Schematics of the different sample surfaces.

Figure S3. Schematic of fabricating through pore on the lotus leaf using a needle.

Figure S4. Schematic of the self-made condensation platform to verify whether the condensed droplets could generate the droplet jetting.

Figure S5. The image, SEM images, and EDS of superhydrophobic surface and superhydrophilic through pore.

Figure S6. The generated processes of the condensed droplets on superhydrophobic Al sheet with a superhydrophilic through pore with 300 μm and four superhydrophilic points with 100 μm.

Figure S7. The coalescence processes of the condensed droplets on superhydrophilic point with the condensed droplets on superhydrophobic Al sheet.

Figure S8. The coalescence processes of the condensed droplets on superhydrophilic point with the surrounding condensed droplets.

Figure S9. Schematic of relationship between the cold plate, sample platform, and water.

Figure S10. The SEM images of the different pore diameters.

Figure S11. The SEM images of the different pore shapes.

Figure S12. The droplet jetting processes and the water collection rate of the different superhydrophilic pore shapes.

Figure S13. The SEM images of the SCA.

Figure S14. Schematic of the different surface refreshing on the SCA.

Figure S15. Schematic of the maximum droplet shedding size on the SCA.

Figure S16. The variation of the droplet coverage ratio on the SCA with the time at different sample surface temperatures.

Figure S17. The condensation processes on the SCA at different relative ambient humidities.

Figure S18. The variation of the droplet coverage ratio on the SCA with the time at different relative ambient humidities.

Figure S19. The variations of the droplet number on the SCA with time at different sample temperatures and different relative ambient humidities.

Figure S20. Schematic of the water collection top wall/ side wall/ bottom wall on the SCA.

Figure S21. The water collection processes of the SHI, SHP, SSHP, SLIPS, and SCA at the side wall.

Figure S22. The water collection processes of the SHI, SHP, SSHP, SLIPS, and SCA at the bottom wall.

Figure S23. Free body diagram of a water droplet on the bottom wall of the SCA.

Figure S24. The images of the condensed droplets on the SHP, SLIPS, and SCA.

Figure S25. The droplet coverage ratio on the different samples.

Figure S26. The water collection rate and the water collection efficiency of the different samples.

Figure S27. Images of the collected water from the SLIPS and the SCA.

Figure S28. UV-Vis-NIR absorption spectra of the water, collected water from SLIPS, and collected water from SCA.

**Tables**

Table S1. The surface subcooling temperature at different sample surface temperatures.

Table S2. The surface subcooling temperature at different relative ambient humidities.

Table S3. The characteristic length of the different samples.

Table S4. Some parameters of the different samples.

Table S5. The water collection efficiency of the different surfaces at the top wall.

Table S6. The water collection efficiency of the different surfaces at the side wall.

Table S7. The water collection efficiency of the different surfaces at the bottom wall.

Table S8. Comparison of the water collection rate of the SCA with that of the reported in the literatures

Table S9. Total organic carbon content in water collected from the SCA and SLIPS

Table S10. Heavy metal ion concentration of the water collected by the SCA and the water from the faucet

**Videos**

Video S1. Droplet jetting phenomenon on a lotus leaf with a pore.

Video S2. Droplet jetting phenomenon of the condensed droplets.

Video S3. Droplet jetting processes at different pore shapes.

Video S4. Surface refreshing I on the SCA in ESEM.

Video S5. Surface refreshing II on the SCA in ESEM.

Video S6. Surface refreshing III on the SCA in ESEM.

Video S7. Dynamic behaviors of condensed droplets on the SCA at different sample temperatures.

Video S8. Dynamic behaviors of condensed droplets on the SCA at different relative ambient humidities.

Video S9. Water collection behavior of different methods at the top wall.

Video S10. Water collection behavior of different methods at the side wall.

Video S11. Water collection behavior of different methods at the bottom wall.

**SI Figures**


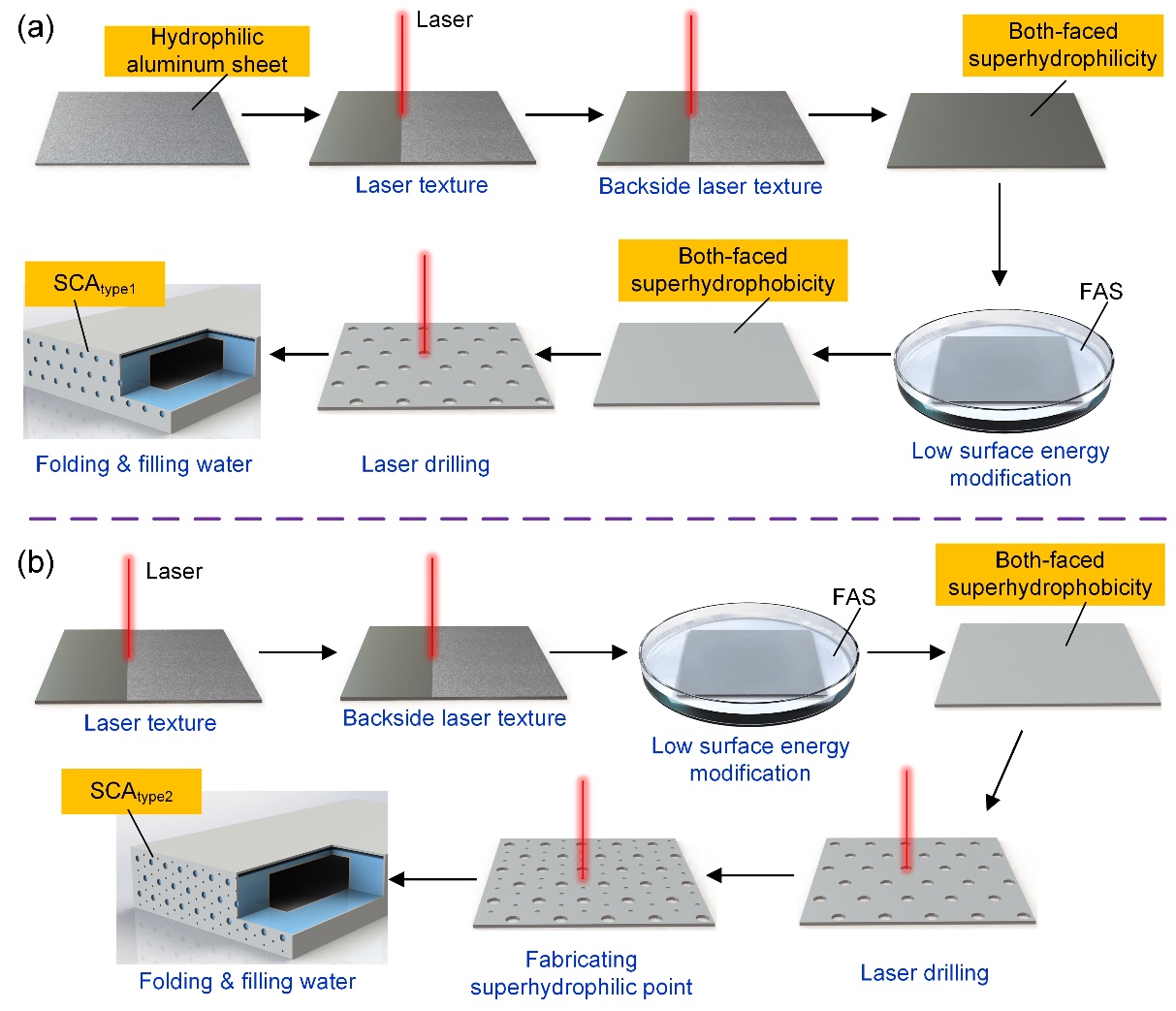


**Figure S1.** Schematics of the fabrication processes of superhydrophobic condensation absorber (SCA) based on droplet jetting phenomenon. (a) Schematics of the fabrication processes of the SCA_type1_. (b) Schematics of the fabrication processes of the SCA_type2_.


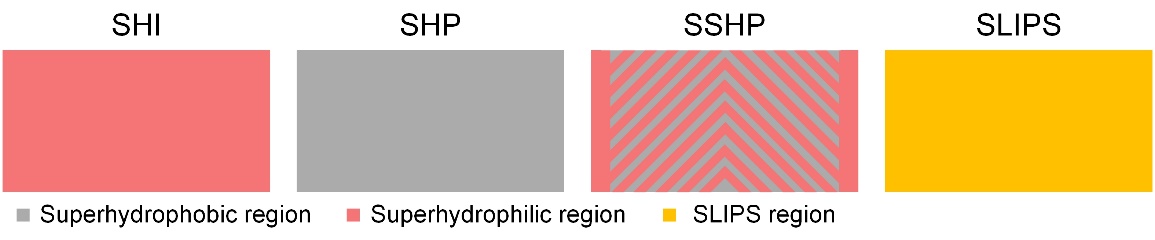


**Figure S2.** Schematics of different sample surfaces, where SHI, SHP, SSHP, and SLIPS meant the entire superhydrophilic surface, entire superhydrophobic surface, superhydrophilic/superhydrophobic hybrid pattern, and slippery liquid-infused porous surface.

*Fabrication of SHI*: The Al sheet was cleaned by sandpaper with 800# and ultrasonic cleaner with acetone for 5 min to remove contaminant. Then, the Al sheet was textured via a nanosecond laser to obtain the SHI.

*Fabrication of SHP*: The above SHI was immersed in 2 wt.% FAS ethanol solution for 60 min and dried at 85 ℃ for 10 min to obtain the SHP.

*Fabrication of SSHP*: We used the laser to process the designed SSHP on SHP to obtain the SSHP.

*Fabrication of SLIPS*: The SHP was dipped into the silicone oil for ~10 min and was then placed vertically in air for ~30 min to remove the excess lubricant and generate a uniform silicon oil film.


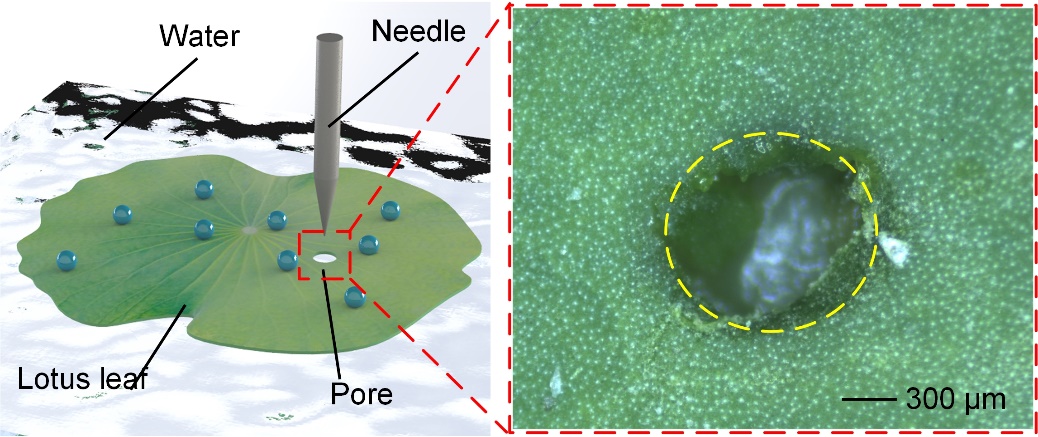


**Figure S3.** Schematic of fabricating through pore on the lotus leaf using a needle. The enlarged image showed the through pore on the lotus leaf.


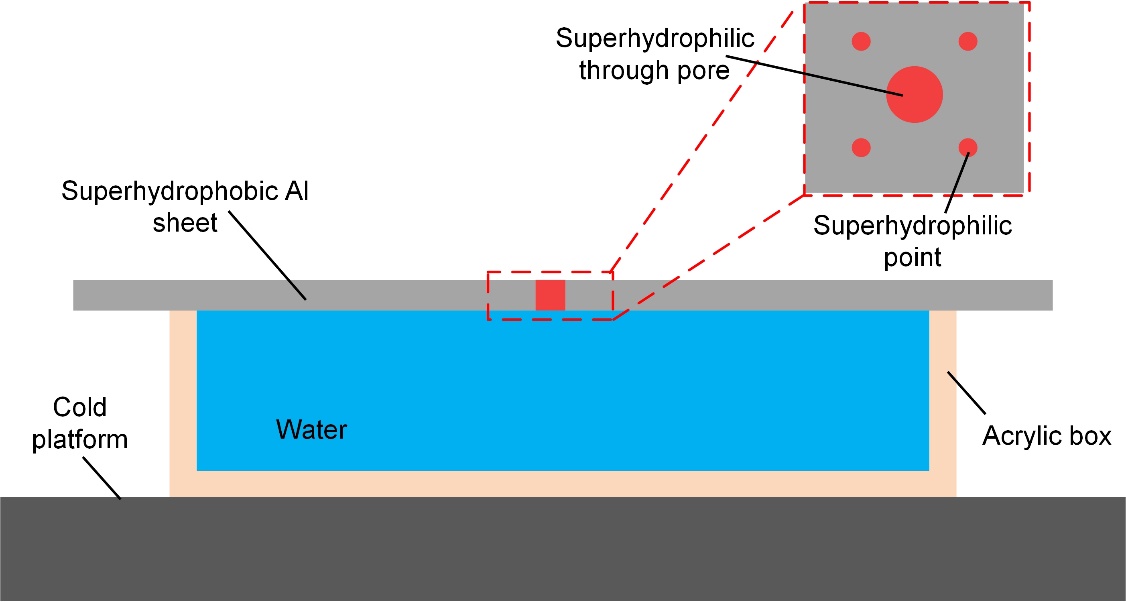


**Figure S4.** Schematic of the self-made condensation platform to verify whether the condensed droplets could generate the droplet jetting.


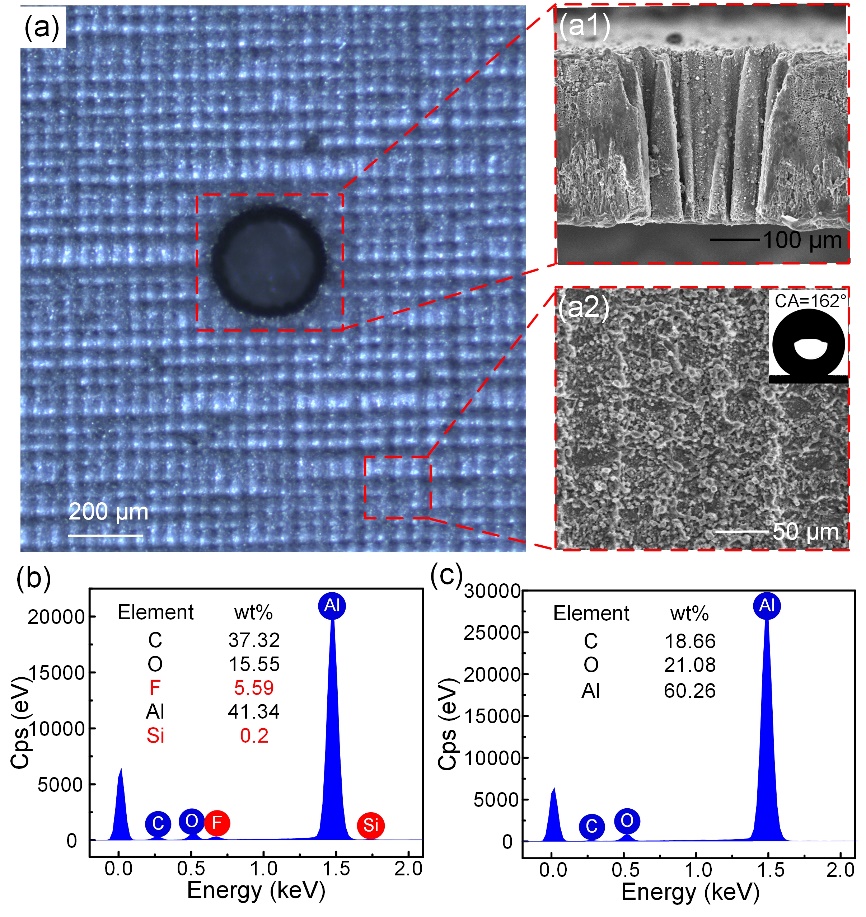


**Figure S5.** The image, SEM images, and EDS of superhydrophobic surface and superhydrophilic through pore. (a) The image of the SCA. (a1) The SEM image of the cross-section of superhydrophilic through pore. (a2) The SEM image of superhydrophobic surface. (b) Superhydrophobic surface after the FAS modification. After modification with the FAS, F and Si element appeared. (c) Superhydrophilic pore after the laser drilling. F and Si elements were not detected on the side wall of superhydrophilic pore after laser drilling.


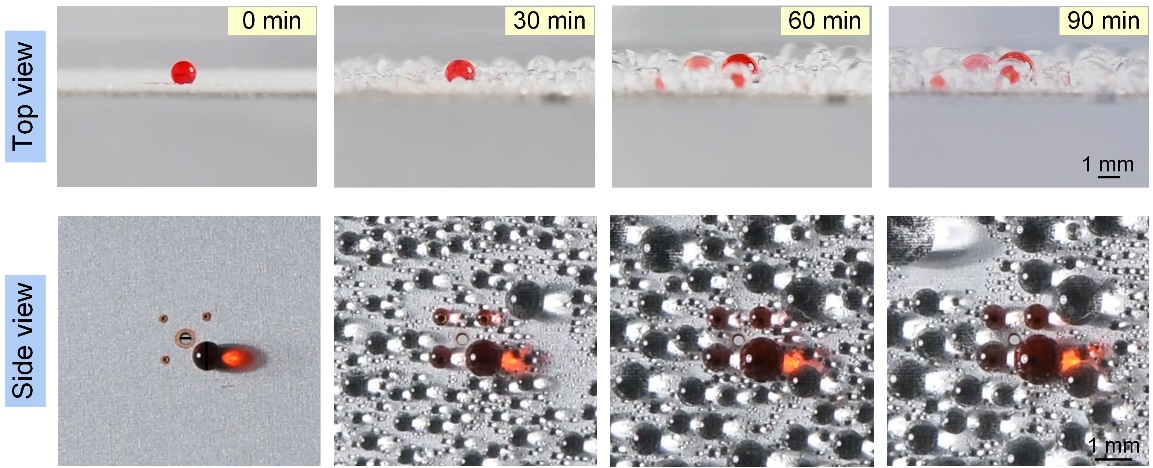


**Figure S6.** The generated processes of the condensed droplets on superhydrophobic Al sheet with a superhydrophilic through pore with 300 μm diameter and four superhydrophilic points with 100 μm diameter, where the ambient temperature, relative ambient humidity, and sample surface temperature for this experiment were 26 ℃ ± 1 ℃, 70% ± 2.5%, and 10 ℃ ± 1 ℃, respectively.


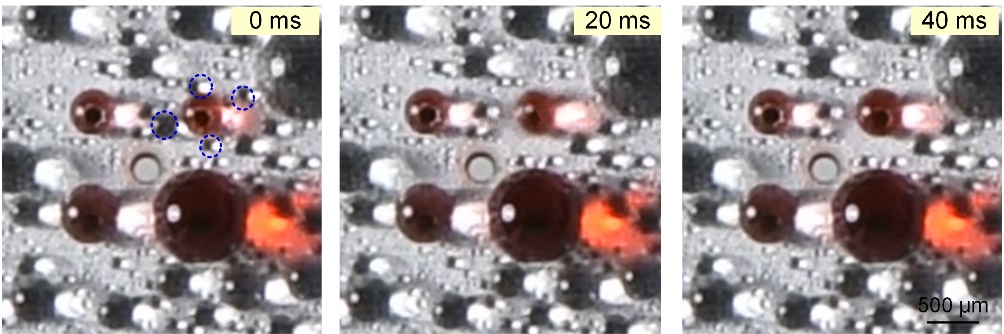


**Figure S7.** The coalescence processes of the condensed droplets on superhydrophilic point with the condensed droplets on superhydrophobic Al sheet.The whole process was about 40 ms.


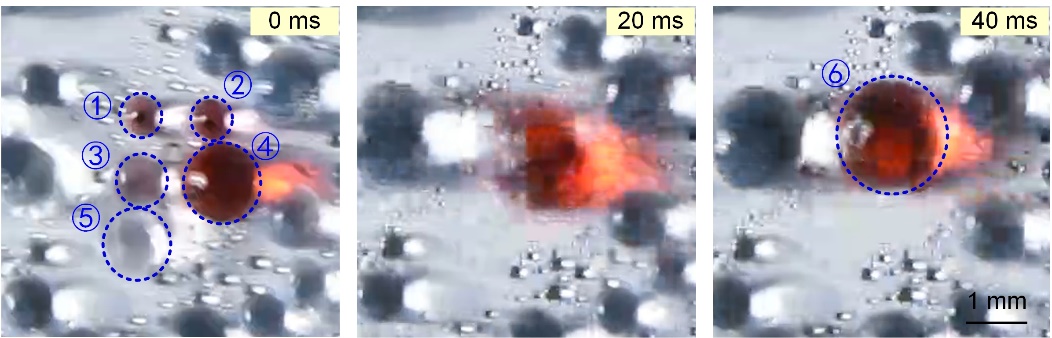


**Figure S8.** The coalescence processes of the condensed droplets on superhydrophilic point with the surrounding condensed droplets.


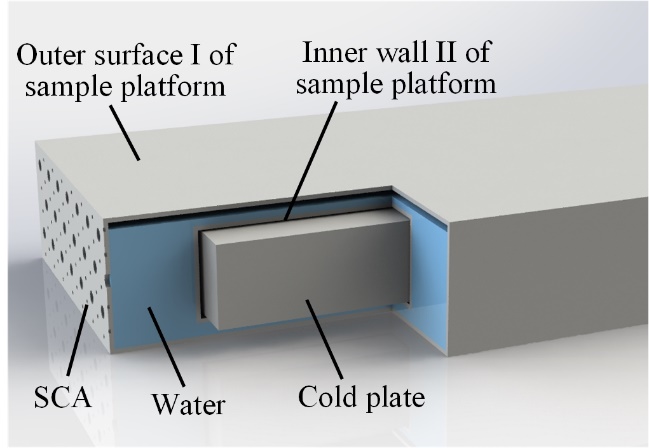


**Figure S9.** Schematic of relationship between the cold plate, sample platform, and water.


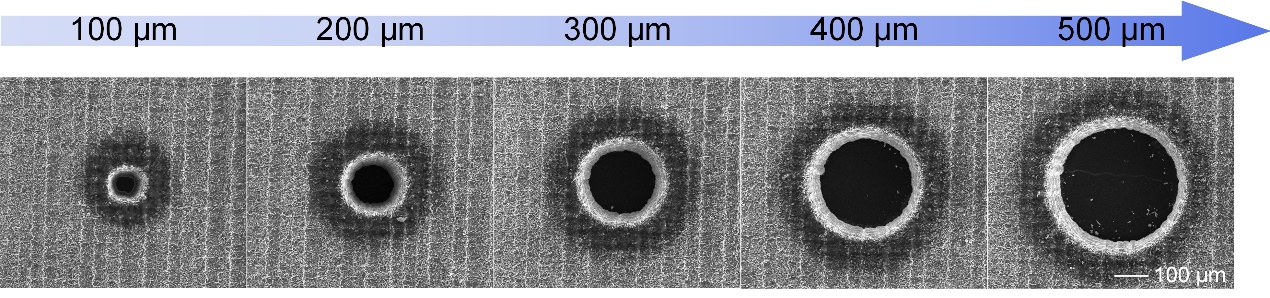


**Figure S10.** The SEM images of the different pore diameters.


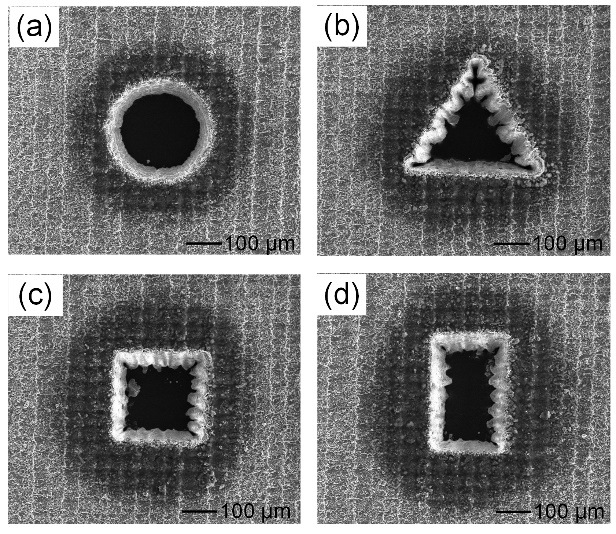


**Figure S11.** The SEM images of the different pore shapes.


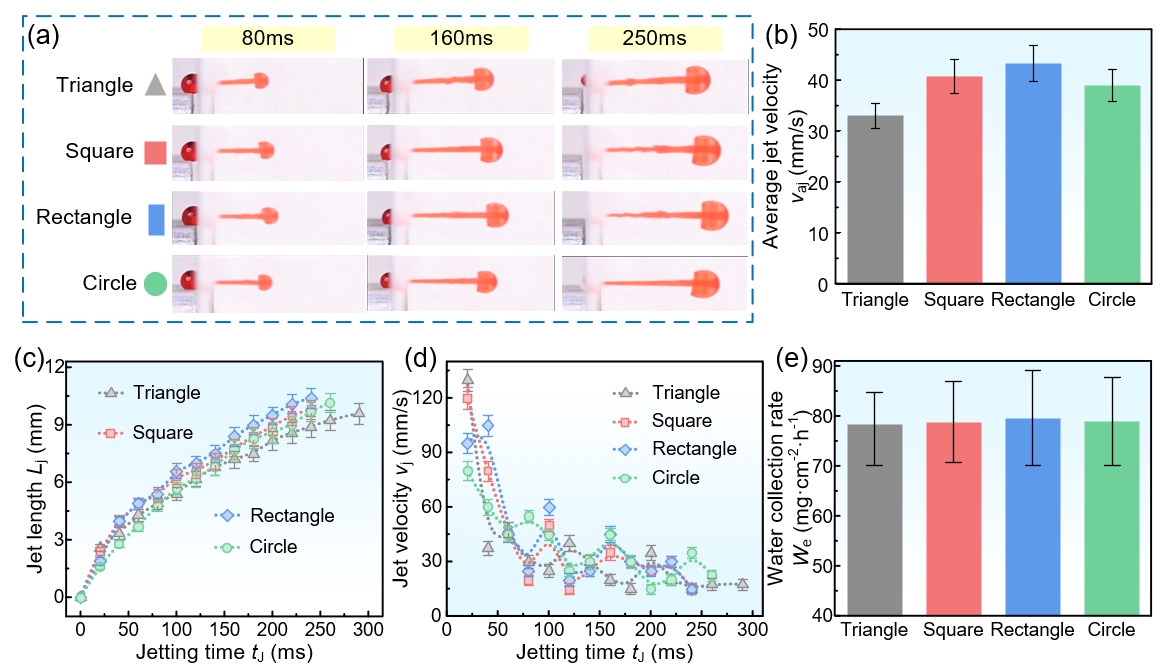


**Figure S12.** The droplet jetting processes and the water collection rate of the different superhydrophilic pore shapes. (a) The droplet jetting processes images of the different superhydrophilic pore shapes. (b) The average jet velocity of the different superhydrophilic pore shapes. (c) The jet length of the different superhydrophilic pore shapes. (d) The jet velocity of the different superhydrophilic pore shapes. (e) The water collection rate of the different superhydrophilic pore shapes. The pore area was about 0.07 mm^2^. The superhydrophilic rectangular pore had a higher average jet velocity compared with that of the circle pore, but the water collection rate of rectangular pore was almost the same as that of circle pore. If the SCA needs be fabricated for a large-scale in the future, the fabrication difficulty of rectangular pore will be much higher than that of circle pore. Therefore, we chose the circle pore for subsequent water collection research.


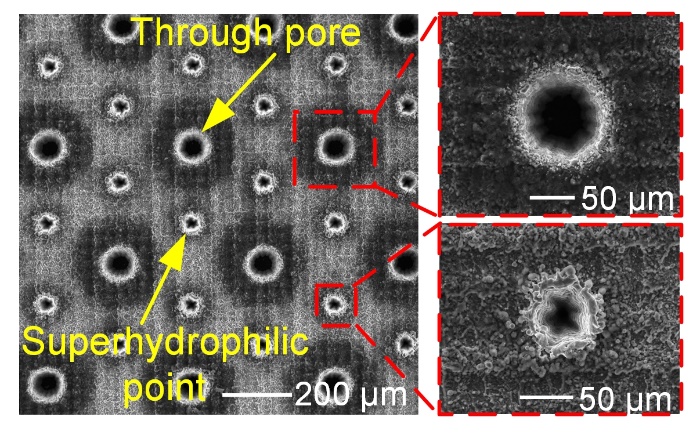


**Figure S13.** The SEM images of the SCA. The diameter of superhydrophilic pore was 100 μm. The diameter of superhydrophilic point was 50 μm. In the study of the influence of the SCA structure parameters on the water collection rate, the diameter range of superhydrophilic pore *d*_p_ was 100 μm to 500 μm because that the *d*_p_ larger than 500 μm would easily cause water leakage and the *d*_p_ less than 100 μm was difficult to fabricate through pore. When the *d*_p_ was 100μm, the diameter of the superhydrophilic region around the pore was about 300 μm. Therefore, we chose the 300 μm as the minimum space between superhydrophilic region edges. The space *S*_e_ range between superhydrophilic region edges was 300 μm to 1500 μm. The spot size of this laser was 50 μm, so we chose the 50 μm as the minimum diameter of superhydrophilic point. The diameter *d*_sp_ of superhydrophilic point was 50 μm to 150 μm. The parameter optimization experiments were conducted within the *d*_p_ of 100 μm to 500 μm, the *S*_e_ of 300 μm to 1500 μm, the *d*_sp_ of 50 μm to 150 μm. Ultimately, we chose the *d*_p_ of 100 μm, the *S*_e_ of 300 μm, and the *d*_sp_ of 50 μm as the structure parameters of the SCA.


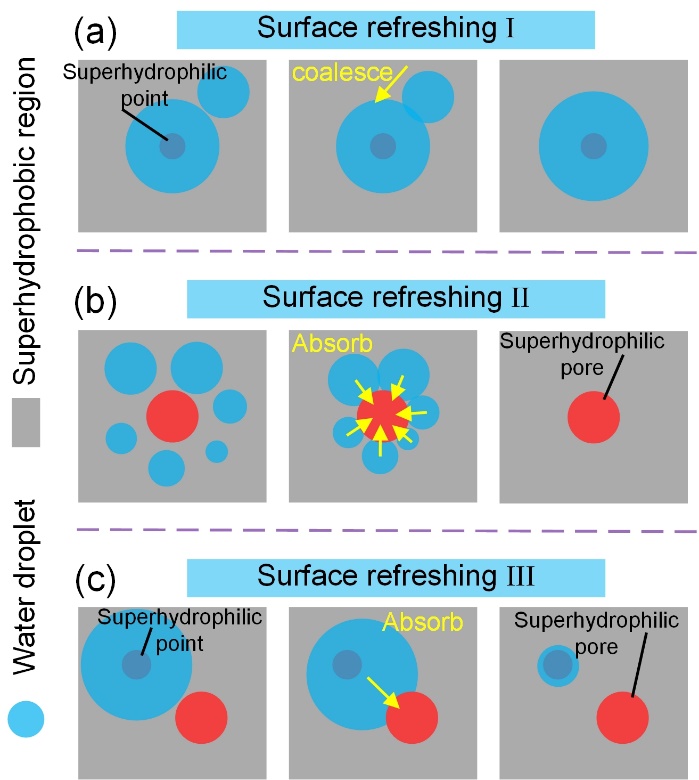


**Figure S14.** Schematic of the different surface refreshing on the SCA. (a) The condensed droplet on superhydrophobic region was coalseced by the droplet on superhydrophilic point, resulting in refreshing surface around this superhydrophilic point. (b) The condensed droplets on superhydrophobic region were absorbed in superhydrophilic pore, resulting in refreshing surface around this superhydrophilic pore. (c) The condensed droplet on superhydrophilic point was absorbed in superhydrophilic pore, resulting in refreshing surface around this superhydrophilic pore.


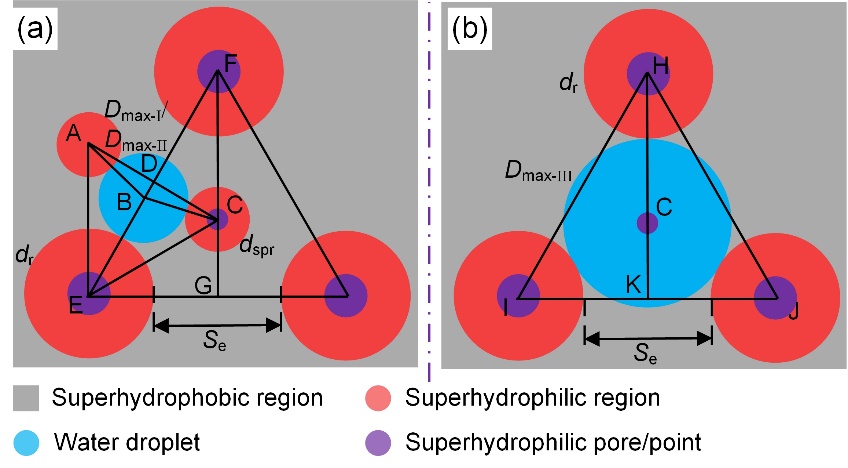


**Figure S15.** Schematic of the maximum droplet shedding size on the SCA. (a) Schematic of the maximum droplet shedding size of the surface refreshing I and surface refreshing II on the SCA. (b) Schematic of the maximum droplet shedding size of the surface refreshing III on the SCA. Superhydrophilic region around superhydrophilic point was *d*_spr_, Superhydrophilic region around superhydrophilic pore was *d*_r_, and the space between superhydrophilic region around the pore edges was *S*_e_.

In the Figure S14(a), according to geometric relationship, it can be seen that the diameter of the blue circle is the maximum droplet shedding size of the surface refreshing I *D*_max-I_ and surface refreshing II *D*_max-II_. In the triangle ABC, it can be obtained,

$\cos\angle\text{BAC}=\frac{\text{AB}^{2}+\text{AC}^{2}-\text{BC}^{2}}{2\times\text{AB}\times\text{AC}}$ (S1)

where $\text{AB}=\text{BC}=D_{\max-\text{I}}+\frac{d_{\text{spr}}}{2}$, $\text{AC}=\text{EC}=\frac{\sqrt{3}\left(S_{\text{e}}+d_{\text{r}}\right)}{3}$. In the triangle ABD, it can be obtained,

$\cos\angle\text{BAD}=\frac{\text{AB}^{2}+\text{AD}^{2}-\text{BD}^{2}}{2\times\text{AB}\times\text{AD}}$ (S2)

where $\text{AD}=\frac{\text{AC}}{2}=\frac{\sqrt{3}\left(S_{\text{e}}+d_{\text{r}}\right)}{6}$, . According to geometric relationship, $\angle\text{BAC}=\angle\text{BAD}$. Combined with Equations (S1) and (S2), we can be obtained,

$\frac{\text{AB}^{2}+\text{AC}^{2}-\text{BC}^{2}}{2\times\text{AB}\times\text{AC}}=\frac{\text{AB}^{2}+\text{AD}^{2}-\text{BD}^{2}}{2\times\text{AB}\times\text{AD}}$ (S3)

after simplification, the *D*_max-I_ or the *D*_max-II_ was expressed as,

$D_{\text{max-I}}=D_{\text{max-II}}=\frac{\left(S_{\text{e}}+d_{\text{r}}\right)^{2}+3\left(S_{\text{e}}\right)^{2}-3\left(d_{\text{spr}}\right)^{2}}{6\left(S_{\text{e}}+d_{\text{spr}}\right)}$ (S4)

where *S*_e_, *d*_r_, *d*_spr_ are 300 μm, 300 μm and 150 μm. Therefore, the *D*_max-I_ or the *D*_max-II_ can be calculated by Equations (S4), which is 208 μm.

In the Figure S14(a), the diameter of the blue circle is the maximum droplet shedding size of the surface refreshing III *D*_max-III_. In the triangle HKJ, $\text{HK}=\frac{\sqrt{3}}{2}\text{HJ}=\frac{\sqrt{3}}{2}\left(S_{\text{e}}+d_{\text{r}}\right)$,

$\text{CH}=\frac{2}{3}\text{HK}=D_{\text{max-III}}+d_{\text{r}}$ (S5)

after simplification, the *D*_max-III_ was expressed as,

$D_{\text{max-III}}=\frac{\left(2\sqrt{3}-3\right)d_{\text{r}}+2\sqrt{3}S_{\text{e}}}{3}$ (S6)

where *S*_e_ and *d*_r_ are 300 μm and 300 μm. Therefore, the *D*_max-III_ can be calculated by Equations (S6), which is 393 μm.


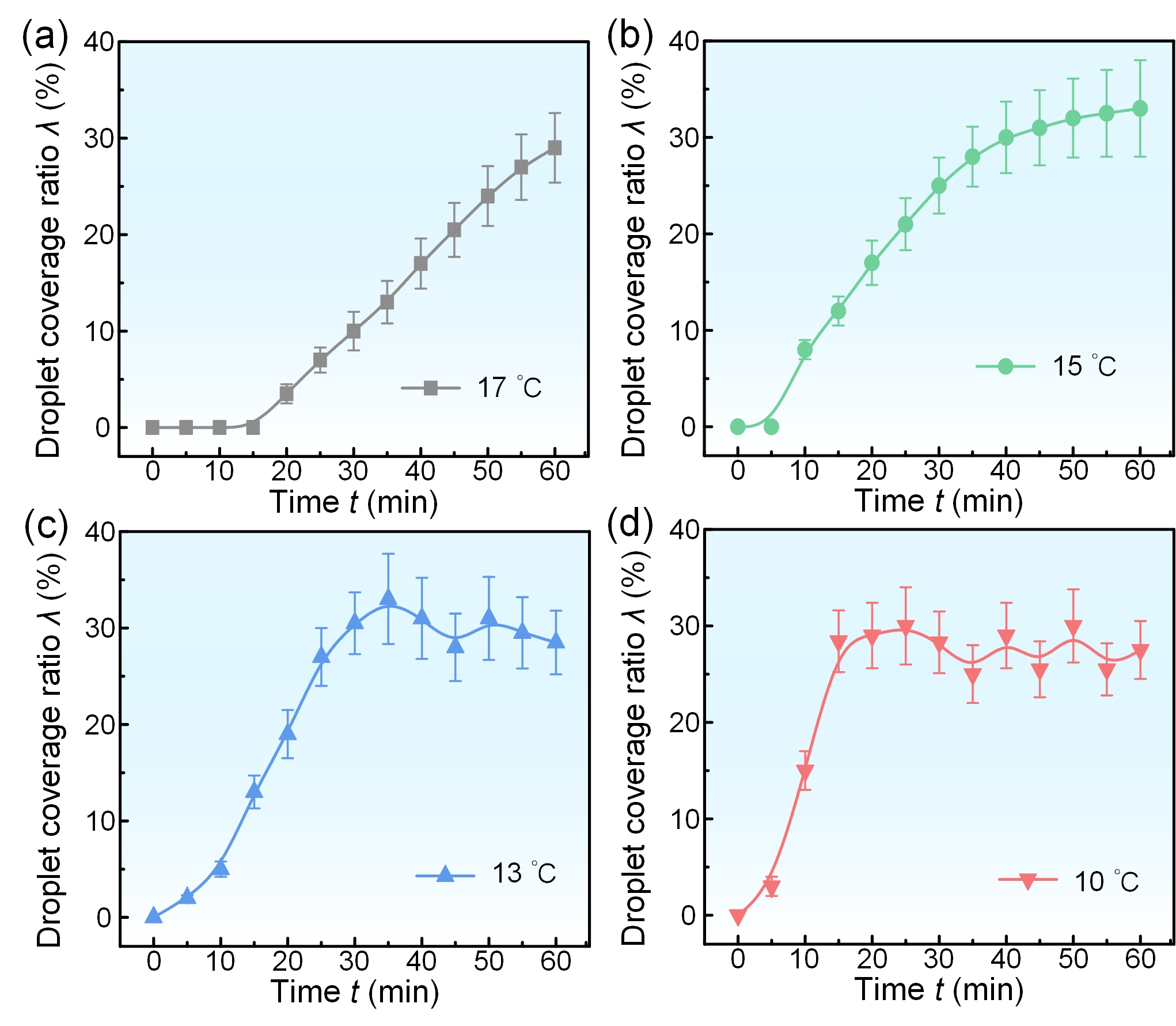


**Figure S16.** The variation of the droplet coverage ratio on the SCA with the time at different sample surface temperatures. (a) The variation of the droplet coverage ratio on the SCA with 17 ℃ sample surface temperature. (b) The variation of the droplet coverage ratio on the SCA with 15 ℃ sample surface temperature. (c) The variation of the droplet coverage ratio on the SCA with 13 ℃ sample surface temperature. (d) The variation of the droplet coverage ratio on the SCA with 10 ℃ sample surface temperature.


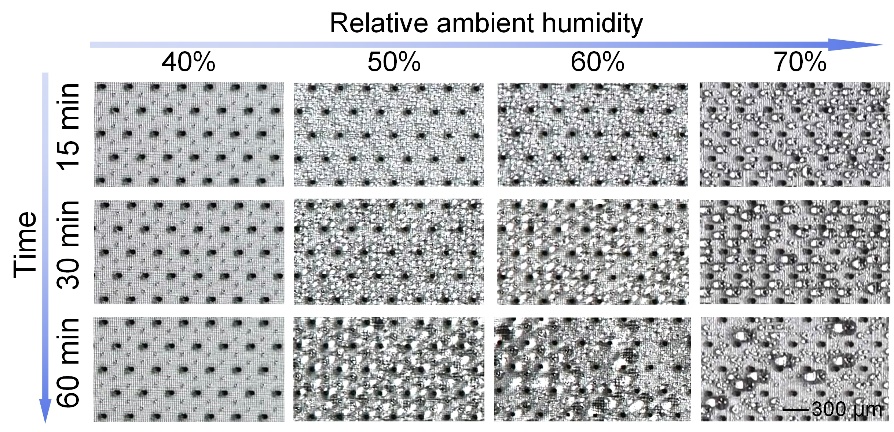


**Figure S17.** The condensation processes on the SCA at different relative ambient humidities, where the ambient temperature and the sample surface temperature were 26 ℃ ± 1 ℃ and 10 ℃ ± 1 ℃


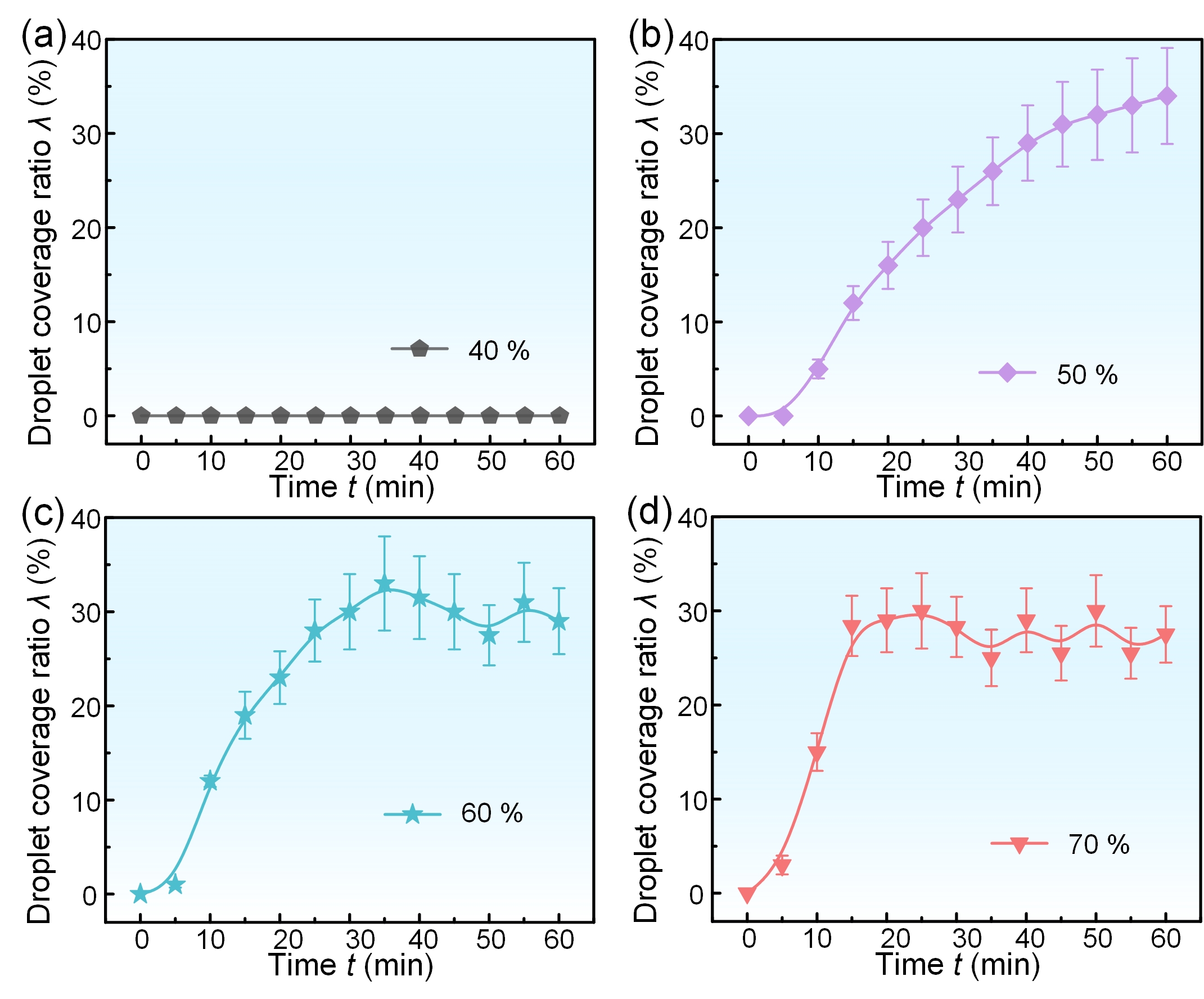


**Figure S18.** The variation of the droplet coverage ratio on the SCA with the time at different relative ambient humidities. (a) The variation of the droplet coverage ratio on the SCA with 40% relative ambient humidity. (b) The variation of the droplet coverage ratio on the SCA with 50% relative ambient humidity. (c) The variation of the droplet coverage ratio on the SCA with 60% relative ambient humidity. (d) The variation of the droplet coverage ratio on the SCA with 70% relative ambient humidity.


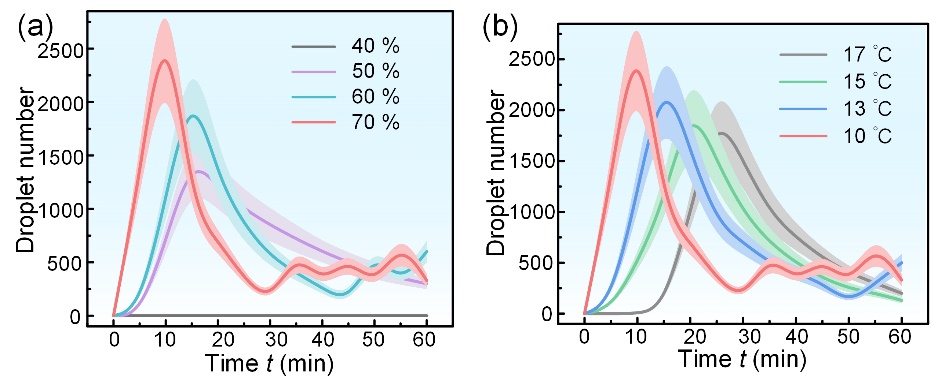


**Figure S19.** The variations of the droplet number on the SCA with time at different relative ambient humidities and different sample surface temperatures. (a) The variations of the droplet number on the SCA with time at different relative ambient humidities, where the ambient temperature and the sample surface temperature were 26 ℃ ± 1 ℃ and 10 ℃ ± 1 ℃. (b) The variations of the droplet number on the SCA with time at different sample surface temperatures, where the ambient temperature and the relative ambient humidity were 26 ℃ ± 1 ℃ and 70% ± 2.5%.


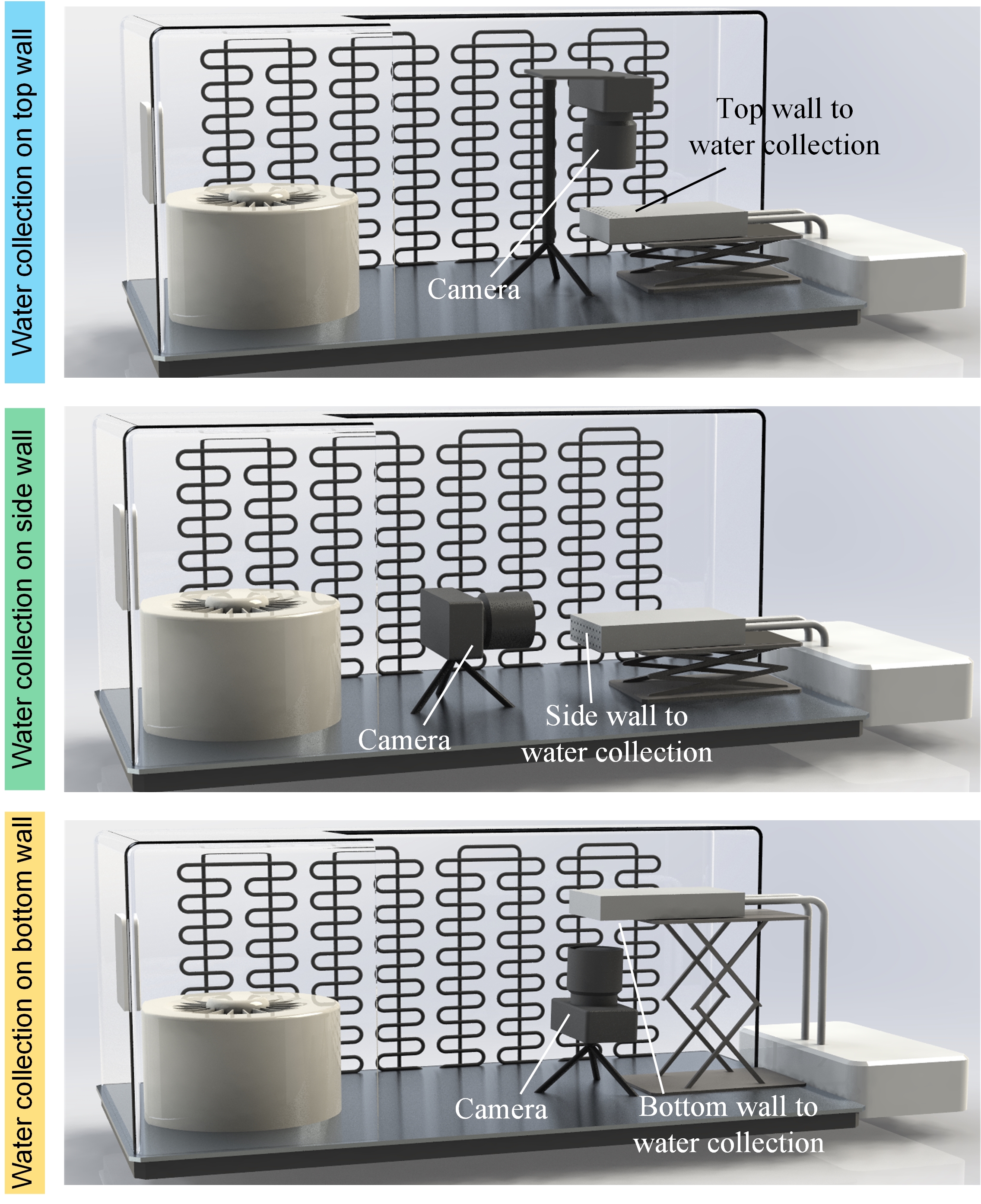


**Figure S20.** Schematic of the water collection top wall/ side wall/ bottom wall on the SCA.

**
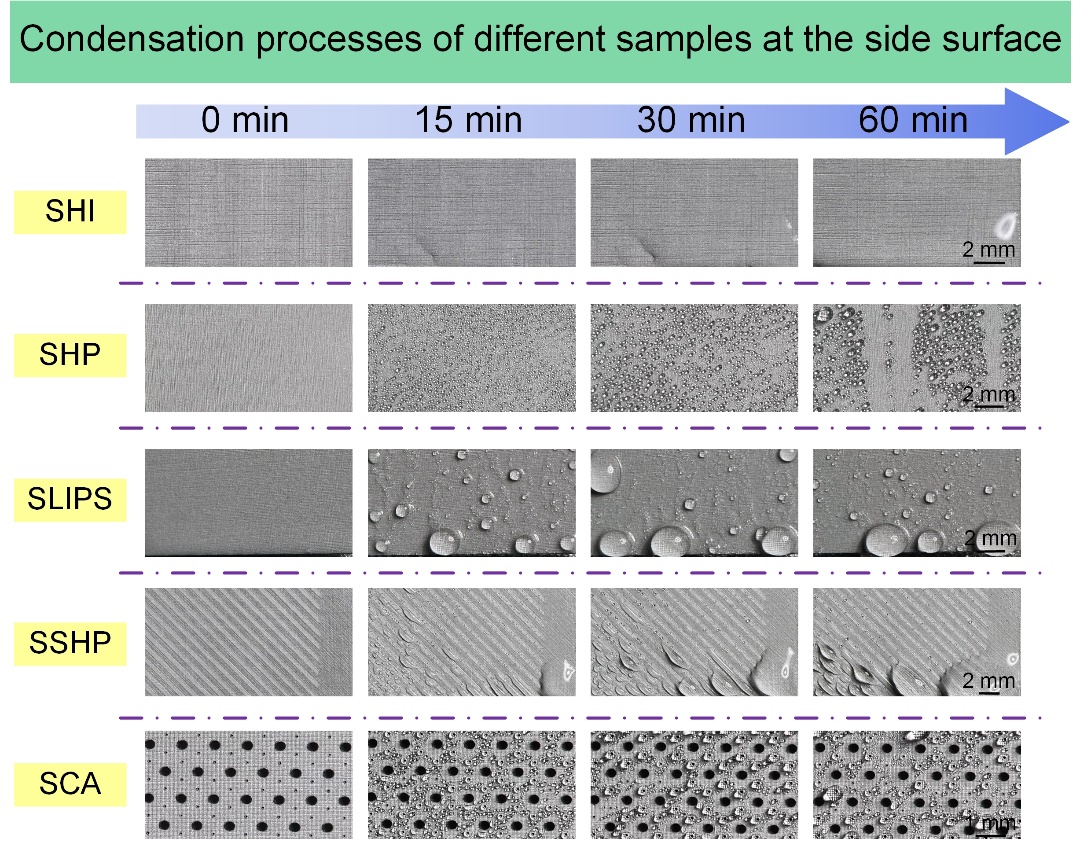
**

**Figure S21.** The water collection processes of the SHI, SHP, SSHP, SLIPS, and SCA at the side wall. In these water collection experiments, the ambient temperature, relative ambient humidity, and sample surface temperature for this experiment were 26 ℃ ± 1 ℃, 70% ± 2.5%, and 10 ℃ ± 1 ℃, respectively.


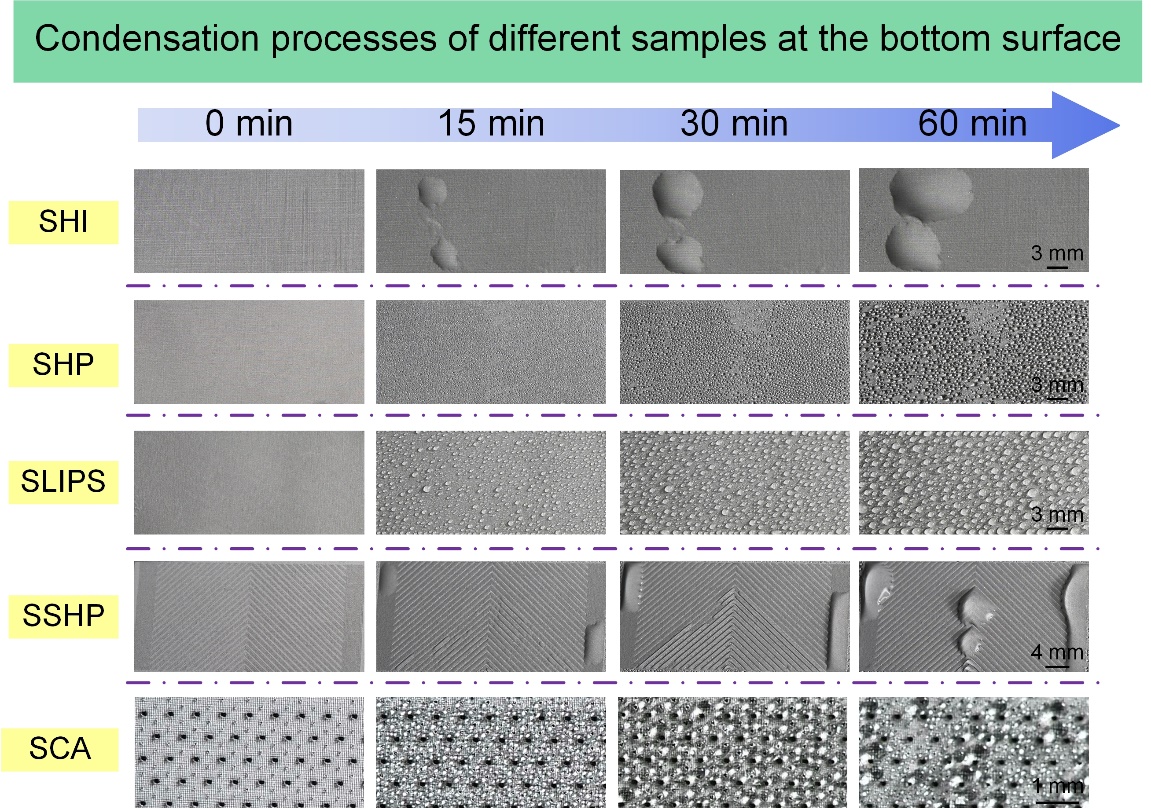


**Figure S22.** The water collection processes of the SHI, SHP, SSHP, SLIPS, and SCA at the bottom wall. In these water collection experiments, the ambient temperature, relative ambient humidity, and sample surface temperature for this experiment were 26 ℃ ± 1 ℃, 70% ± 2.5%, and 10 ℃ ± 1 ℃, respectively.


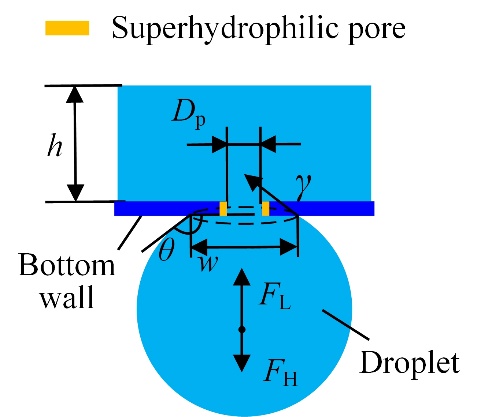


**Figure S23.** Free body diagram of a water droplet on the bottom wall of the SCA. At the bottom wall, the droplet should against gravity to enter the SCA. However, the shedding droplets diameter was very small (about 300 μm), which would generate significant Laplace force. As shown in Figure R5, a water droplet is on the pore of the bottom wall, which is subjected to the Laplace force *F*_L_ from Laplace pressure *P*_L_ and the resistance force *F*_H_ from hydrostatic pressure *P*_H_ in the vertical direction. The Laplace pressure *P*_L_ was calculated by

$P_{\text{L}}=\frac{4\gamma\cos\left(\theta-90^{\circ}\right)}{w}$ (S7)

where *γ*, *w*, and *θ* are the water surface tension which is ~72.8 mN·m^-1^, the contact line width, and the water contact angle, respectively. Then, the Laplace force *F*_L_ from the Laplace pressure was given

$F_{\text{L}}=P_{\text{L}}A_{\text{pore}}=\frac{\pi\gamma D_{\text{p}}^{2}\cos(\theta-90^{\circ})}{w}$ (S8)

The pore area *A*_pore_ can be calculated by *A*_pore_=π(*D*_p_/2)^2^, where *D*_p_ is the pore diameter. Since the hydrostatic pressure *P*_H_=*ρgh*, the resistance force *F*_H_ was described as

$F_{\text{H}}=P_{\text{H}}A_{\text{pore}}=\pi\rho gh\left(\frac{D_{\text{p}}}{2}\right)^{2}$ (S8)

where *ρ* is the water density, *g* is the gravitational acceleration, *h* is the water depth. Then, the driving force *F*_d_ was

$F_{\text{d}}=F_{\text{L}}-F_{\text{H}}=\frac{\pi\gamma D_{\text{p}}^{2}\text{cos}\left(\theta-90^{\circ}\right)}{w}-\frac{\pi\rho ghD_{\text{p}}^{2}}{4}$ (S10)

If the driving force is greater than 0, it means that the water droplet can enter the pore of SCA under the Laplace force. Assuming the contact line width of 300 μm and the contact angle of 150°, According to Equation R4, it can be calculated that the water depth is 70 mm, which means that if the water height is not larger than 70 mm, the water in the SCA will not leak.


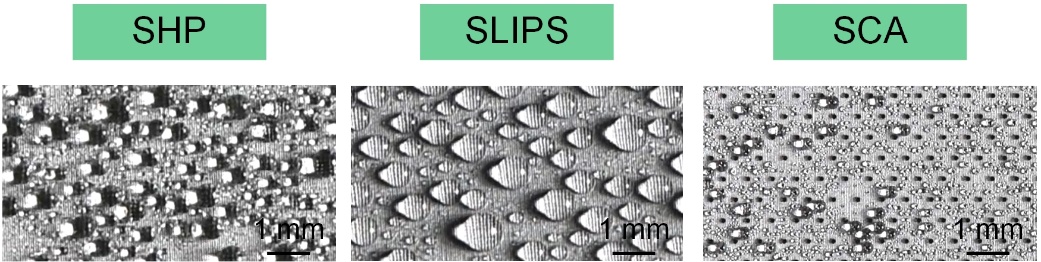


**Figure S24.** The images of the condensed droplets on the SHP, SLIPS, and SCA. The shedding size of the condensed droplets on the SCA was obviously less than that on the SHP and the SLIPS.


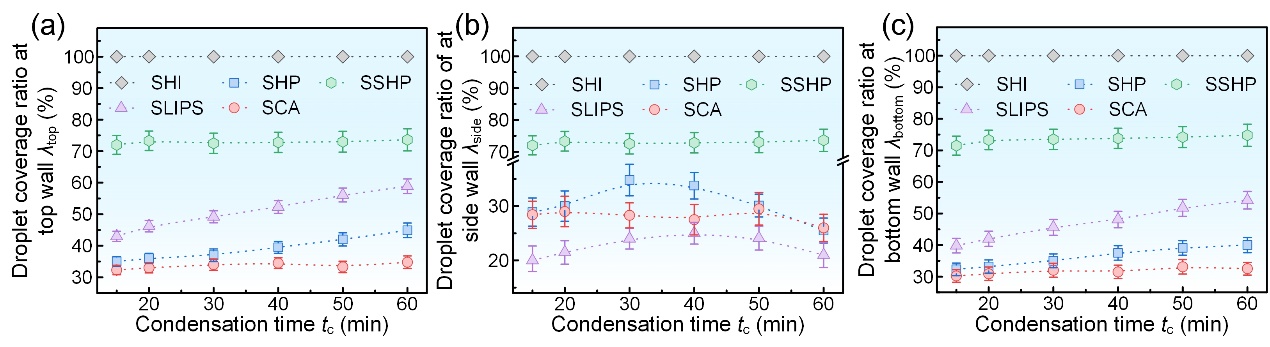


**Figure S25.** The droplet coverage ratio on the different samples. (a) The droplet coverage ratio of the different samples at the top wall. (b) The droplet coverage ratio of the different samples at the side wall. (c) The droplet coverage ratio of the different samples at the bottom wall.


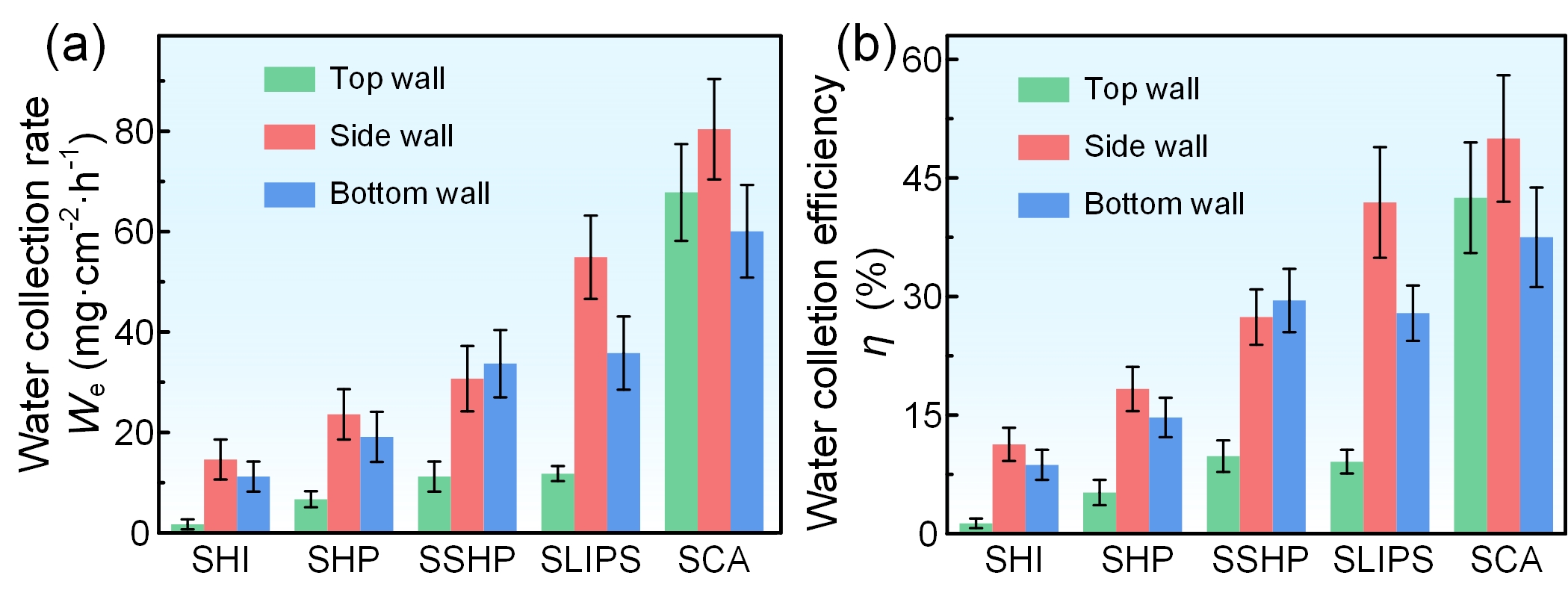


**Figure S26.** The water collection rate and the water collection efficiency of the different samples. (a) The water collection rate of the different samples at the top wall/ side wall/ bottom wall. (b) The water collection efficiency of the different samples at the top wall/ side wall/ bottom wall. In these water collection experiments, the ambient temperature, relative ambient humidity, and sample surface temperature for this experiment were 26 ℃ ± 1 ℃, 70% ± 2.5%, and 10 ℃ ± 1 ℃, respectively.

To objectively evaluate the water collection capability of the different surfaces, we developed a performance figure of merit for this condensation experiment system. The water collection efficiency *η* was used to appraise the water collection capability from the atmosphere, which could be described as follows,

$\eta=\frac{W_{\text{e}}}{W_{\text{t}}}\times100\%$ (S11)

where *W*_e_ and *W*_t_ are the experimental water collection rate and the theoretical water collection rate at the sample surface. The experimental water collection rate was calculated as follows,

$W_{\text{e}}=\frac{m_{\text{e}}}{A_{\text{e}}\times t_{\text{e}}}$ (S12)

where *m*_e_, *A*_e_, and *t*_e_ are the water collection mass, the collection area, and the collection time. The theoretical water collection rate at the sample surface aimed to calculate a value that would be used as a reference value for comparison, rather than for an accurate theoretical estimation of the collected water. In order to calculate the maximum mass transfer during the water collection processes, it was necessary to specify the mass transfer type between the air and the water collection surface, such as the natural convection or the forced convection. During the water collection processes, it was necessary to ensure constant ambient relative humidity by timely adjusting the opening or closing of the humidifier without fog. When the humidifier was turned off, the mass transfer type was the natural convection in the closed chamber. For the ambient temperature was 26 ℃, the Prandtl number *Pr* was expressed as follows,

$Pr=\frac{\mu C}{K}$ (S13)

where *μ*, *C*, and *K* were the dynamic viscosity (18.5×10^-6^ Pa·s), the specific heat (1000 J·kg^-1^·K^-1^), the thermal conductivity (0.0262 W·m^-1^·K^-1^). After calculation, the *Pr* was 0.7. The Grashof number *Gr* could was expressed as follows,

$Gr=\frac{L^{3}\rho^{2}g\Delta T\beta}{\mu^{2}}$ (S14)

where *L*, *ρ*, *g*, Δ*T*, and *β* were the characteristic length, air density (1.177 kg·m^-3^), gravity acceleration (9.8 m·s^-2^), fluid thermal expansion coefficient (0.0034 ℃^-1^). In these water collection experiments, all sample shapes were the rectangle, the characteristic length *L*=2×*a*×*b*×(*a*+*b*)^-1^, where *a* and *b* were the side length of the rectangle (Table S3). Then, the Rayleigh number *Ra* could be described as follows,

$Ra=Gr\times Pr$ (S15)

The Nusselt number *Nu*_n_ for the natural convection could be calculated as follows,

$Nu_{\text{n}}=0.68+\frac{0.67\times Ra^{0.25}}{\left[1+\left(\frac{0.492}{Pr}\right)^{\frac{9}{16}}\right]^{\frac{4}{9}}}$ (S16)

We then calculated the heat transfer coefficient *h*_n_ for natural convection as follows,

$h_{\text{n}}=\frac{Nu_{\text{n}}\times K}{L}$ (S17)

for the different samples, the *h*_n_ of the SHI, SHP, SLIPS was 7.924 W·m^-2^·K^-1^, the *h*_n_ of the SSHP was 7.213 W·m^-2^·K^-1^, the *h*_n_ of the SCA was 9.197 W·m^-2^·K^-1^.

When the humidifier turned on, the mass transfer type was the forced convection in the closed chamber. The wind velocity *v*_w_ of the humidifier was about 0.6 m·s^-1^. The Reynolds number *Re*=*L*×*v*_w_×*c*^-1^, where *c* was air kinematic viscosity (1.59×10^-5^ m^2^·s^-1^). Then, the Nusselt number *Nu*_f_ for the forced convection could be calculated as follows,

$Nu_{\text{f}}=0.664Pr^{\frac{1}{3}}Re^{\frac{1}{2}}$ (S18)

According to the Equations (S13), the heat transfer coefficient *h*_f_ for the forced convection was expressed as follows,

$h_{\text{f}}=\frac{Nu_{\text{f}}\times K}{L}$ (S19)

for the different samples, the *h*_f_ of the SHI, SHP, SLIPS was 21.278 W·m^-2^·K^-1^, the *h*_f_ of the SSHP was 18.427 W·m^-2^·K^-1^, the *h*_f_ of the SCA was 26.369 W·m^-2^·K^-1^. To calculate the theoretical maximum water collection rate, we assumed that the humidifier was in operation throughout the entire water collection process. Then, the mass transfer coefficient of air *W*_m,air_ could be expressed as follows,

$W_{\text{m,air}}=\frac{h_{\text{f}}}{c_{\text{p}}}\left(Le\right)^{-\frac{3}{4}}$ (S20)

where *c*_p_ and *L*_e_ were the specific heat of the air at constant pressure (1000 J·kg^-1^·K^-1^) and the Lewis number (0.87 at 26.85 ℃). For the relative ambient humidity of 70% and the ambient temperature of 26 ℃, the vapor to air mass fraction was 0.0152. To calculate the maximum mass transfer, we assumed that the sample surface was completely dry, which meant the mass ratio of the vapor to air in the air *k*_air-vapor_ and the mass ratio of the vapor to air on the sample surface *k*_sample-vapor_ were 0.0152 and 0. Therefore, theoretical water collection rate *W*_t_ at the sample surface was expressed as follows,

$W_{\text{t}}=W_{\text{m,air}}\times\Delta k_{\text{vapor}}=W_{\text{m,air}}\times\left(k_{\text{air-vapor}}-k_{\text{sample-vapor}}\right)$ (S21)

After calculation, the *W*_t_ of the SHI, SHP, SLIPS was 129 mg·cm^-2^·h^-1^, the *W*_t_ of the SSHP was 112 mg·cm^-2^·h^-1^, the *W*_t_ of the SCA was 160 mg·cm^-2^·h^-1^.


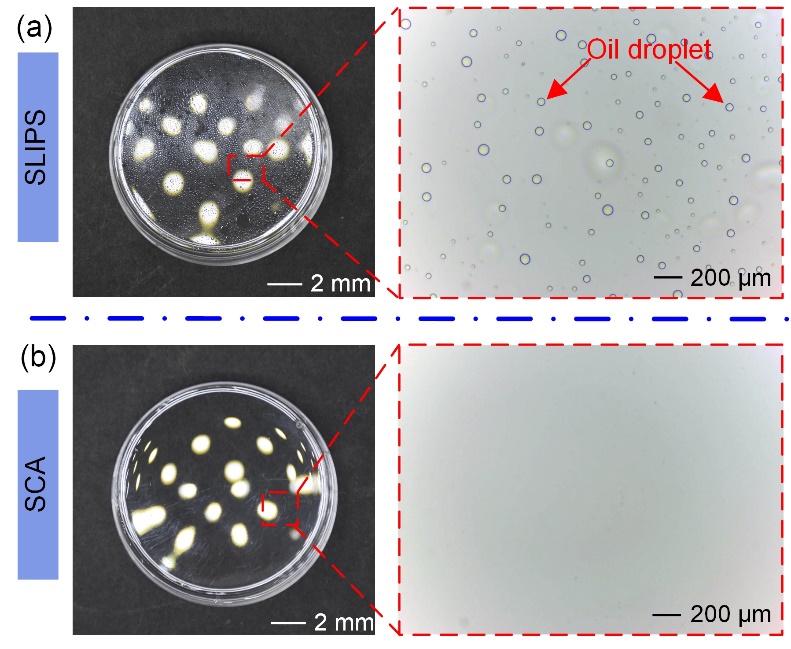


**Figure S27.** Images of the collected water from the SLIPS and the SCA. (a) Images of the collected water from the SLIPS, where the oil droplets could be clearly observed. (b) Images of the collected water from the SCA, where the collected water from the SCA was clean.


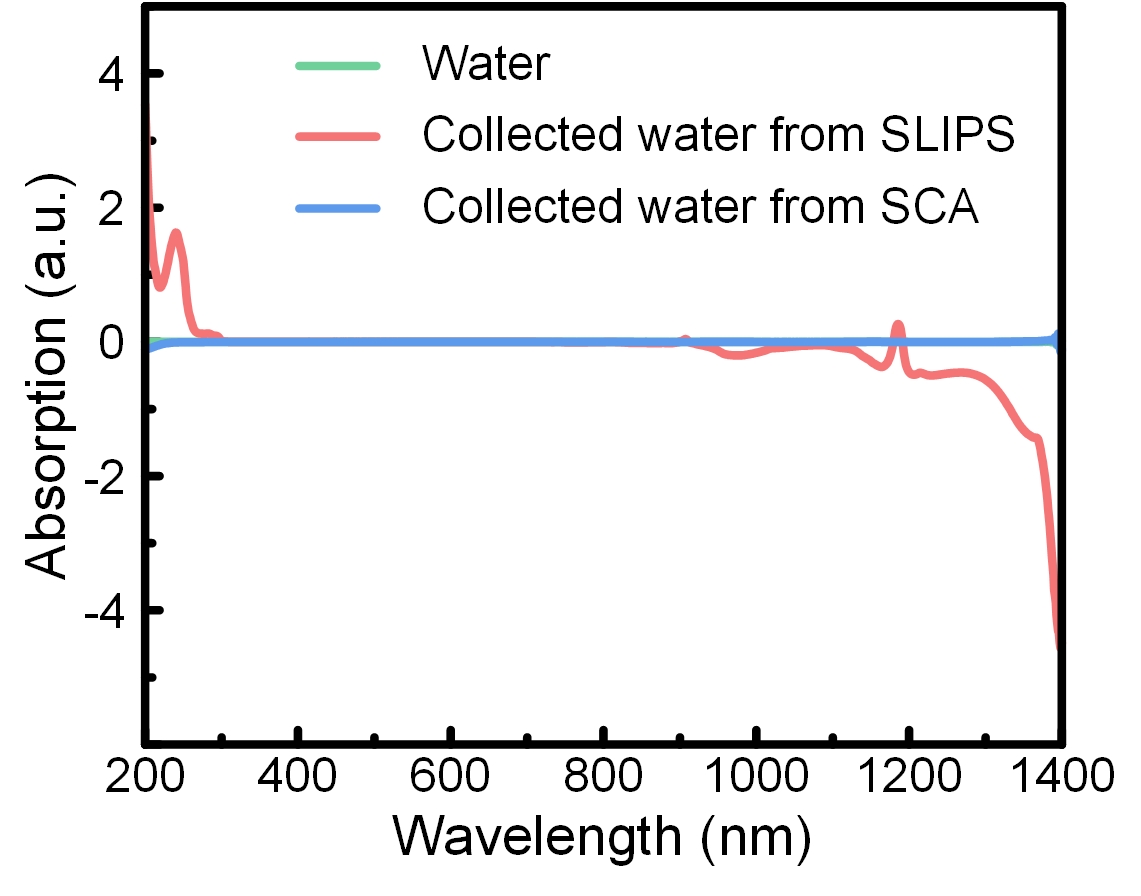


**Figure S28.** UV-Vis-NIR absorption spectra of the water, collected water from SLIPS, and collected water from SCA.

**SI Tables**

**Table S1.** The surface subcooling temperature at different sample surface temperatures.

| Sample surface temperature (℃) | Ambient temperature (℃) | Relative ambient humidity (%) | Dew temperature (℃) | Subcooling temperature (℃) |
| --- | --- | --- | --- | --- |
| 10 | 26 | 70 | 20 | 10 |
| 13 | 26 | 70 | 20 | 7 |
| 15 | 26 | 70 | 20 | 5 |
| 17 | 26 | 70 | 20 | 3 |

**Table S2.** The surface subcooling temperature at different relative ambient humidities.

| Relative ambient humidity (%) | Ambient temperature (℃) | Sample surface temperature (℃) | Dew temperature (℃) | Subcooling temperature (℃) |
| --- | --- | --- | --- | --- |
| 40 | 26 | 10 | 11 | 1 |
| 50 | 26 | 10 | 15 | 5 |
| 60 | 26 | 10 | 18 | 8 |
| 70 | 26 | 10 | 20 | 10 |

**Table S3.** The characteristic length of the different samples.

|  | SHI | SHP | SSHP | SLIPS | SCA |
| --- | --- | --- | --- | --- | --- |
| Long side length *a* (m) | 0.03 | 0.03 | 0.04 | 0.03 | 0.035 |
| Short side length *b* (m) | 0.015 | 0.015 | 0.02 | 0.015 | 0.008 |
| Characteristic length *L* (m) | 0.02 | 0.02 | 0.027 | 0.02 | 0.013 |

**Table S4.** Some parameters of the different samples.

|  | SHI | SHP | SSHP | SLIPS | SCA |
| --- | --- | --- | --- | --- | --- |
| Grashof number *Gr* | 0.02 | 0.02 | 0.027 | 0.02 | 0.013 |
| Rayleigh number *Ra* | 11910.37 | 11910.37 | 28232 | 11910.37 | 3288.47 |
| Nusselt number for natural convection *Nu*_n_ | 6.049 | 6.049 | 7.342 | 6.049 | 4.572 |
| Reynolds number *Re* | 754.72 | 754.72 | 1006.29 | 754.72 | 491.44 |

**Table S5.** The water collection efficiency of the different surfaces at the top wall. In these water collection experiments, the ambient temperature, relative ambient humidity, and sample surface temperature for this experiment were 26 ℃ ± 1 ℃, 70% ± 2.5%, and 10 ℃ ± 1 ℃.

| Sample | Experimental water collection rate  *W*_e_ (mg·cm^-2^·h^-1^) | Theoretical water collection *W*_t_ (mg·cm^-2^·h^-1^) | Water collection efficiency on the top wall  *η*_top_ (%) |
| --- | --- | --- | --- |
| SHI | 1.7 | 129 | 1.3 |
| SHP | 6.7 | 129 | 5.2 |
| SSHP | 11.0 | 112 | 9.8 |
| SLIPS | 11.8 | 129 | 9.1 |
| SCA | 68.0 | 160 | 42.5 |

**Table S6.** The water collection efficiency of the different surfaces at the side wall. In these water collection experiments, the ambient temperature, relative ambient humidity, and sample surface temperature for this experiment were 26 ℃ ± 1 ℃, 70% ± 2.5%, and 10 ℃ ± 1 ℃.

| Sample | Experimental water collection rate  *W*_e_ (mg·cm^-2^·h^-1^) | Theoretical water collection *W*_t_ (mg·cm^-2^·h^-1^) | Water collection efficiency on the top wall  *η*_side_ (%) |
| --- | --- | --- | --- |
| SHI | 14.6 | 129 | 11.3 |
| SHP | 23.6 | 129 | 18.3 |
| SSHP | 30.7 | 112 | 27.4 |
| SLIPS | 54.0 | 129 | 41.9 |
| SCA | 80.0 | 160 | 50.0 |

**Table S7.** The water collection efficiency of the different surfaces at the bottom wall. In these water collection experiments, the ambient temperature, relative ambient humidity, and sample surface temperature for this experiment were 26 ℃ ± 1 ℃, 70% ± 2.5%, and 10 ℃ ± 1 ℃.

| Sample | Experimental water collection rate  *W*_e_ (mg·cm^-2^·h^-1^) | Theoretical water collection *W*_t_ (mg·cm^-2^·h^-1^) | Water collection efficiency on the top wall  *η*_bottom_ (%) |
| --- | --- | --- | --- |
| SHI | 11.2 | 129 | 8.7 |
| SHP | 19.0 | 129 | 14.7 |
| SSHP | 33.0 | 112 | 29.5 |
| SLIPS | 36.0 | 129 | 27.9 |
| SCA | 60.0 | 160 | 37.5 |

**Table S8.** Comparison of the water collection rate of the SCA with that of the reported in the literatures.

| Water collection methods | References | Subcooling temperature (℃) | Experimental water collection rate  *W*_e_ (mg·cm^-2^·h^-1^) |
| --- | --- | --- | --- |
| SCA | This work | 10 | 80 |
| SHP | [1] | 10 | 5.2 |
|  | [2] | 12 | 16 |
|  | [3] | 12.5 | 21.8 |
|  | [4] | 16 | 19 |
|  | [5] | 27 | 75 |
| SSHP | [6] | 10 | 21.5 |
|  | [7] | 12 | 18.22 |
|  | [8] | 13.5 | 25 |
|  | [9] | 14 | 60 |
|  | [10] | 17 | 24 |
| SLIPS | [11] | 11.7 | 3.5 |
|  | [12] | 16 | 13.2 |
|  | [13] | 16 | 43.3 |
|  | [14] | 22 | 60 |

**Table S9.** Total organic carbon content in water collected from the SCA and SLIPS

|  | Total organic carbon content (mg·L^-1^) |
| --- | --- |
| Water collected by the SCA | <0.1 |
| Water collected by the SLIPS | 16.65 |

**Table S10.** Heavy metal ion concentration of the water collected by the SCA and the water from the faucet

|  | Pb (mg·L^-1^) | Cd (mg·L^-1^) | Hg (mg·L^-1^) | Cr (mg·L^-1^) |
| --- | --- | --- | --- | --- |
| Water collected by the SCA | 0.004 | 0.016 | <0.001 | 0.010 |
| Water from the faucet | 0.003 | 0.015 | <0.001 | 0.012 |

**SI Videos**

**Video S1**. Droplet jetting phenomenon on a lotus leaf with a pore. The volume *V*_d_ of the water droplet was 15 μL.

**Video S2**. Droplet jetting phenomenon of the condensed droplets. The ambient temperature (*T*_a_), relative ambient humidity (RH), and sample surface temperature (*T*_s_) for this experiment were 26 ℃ ± 1 ℃, 70% ± 2.5%, and 10 ℃ ± 1 ℃.

**Video S3**. Droplet jetting processes at different pore shapes. The initial droplet volume *V*_0_ was 3 μL. The area *S*_p_ of pore was 0.07 mm^2^.

**Video S4**. Surface refreshing I on the SCA in ESEM.

**Video S5**. Surface refreshing II on the SCA in ESEM.

**Video S6**. Surface refreshing III on the SCA in ESEM.

**Video S7**. Dynamic behaviors of condensed droplets on the SCA at different sample temperatures.

**Video S8**. Dynamic behaviors of condensed droplets on the SCA at different relative ambient humidities.

**Video S9**. Water collection behavior of different methods at the top wall.

**Video S10**. Water collection behavior of different methods at the side wall.

**Video S11**. Water collection behavior of different methods at the bottom wall.

**SI Reference**

[1] I. Haechler, H. Park, G. Schnoering, T. Gulich, MRohner, A. Tripathy, A. Milionis, TM. Schutzius, D. Poulikakos, *Sci. Adv.* **2021**, *7*, eabf3978.

[2] A. Lee, MW. Moon, H, Lim, WD, Kim, HY, Kim, *Langmuir* **2012**, *28*, 10183-10191.

[3] D. Nioras, K. Ellinas, V. Constantoudis, E. Gogolides, *ACS Appl. Mater. Interfaces* **2021**, *13*, 48322-48332.

[4] Z. Guo, D. Monga, L. Shan, D. Boylan, X. Dai, *Droplet* **2022**, *1*, 170-181.

[5] K. Gerasopoulos, W. Luedeman, E. Olceroglu, M. McCarthy, JJ. Benkoski, *ACS Appl. Mater. Interfaces* **2018**, *10*, 4066-4076.

[6] A. Rajabi-Abhari, M. Soltani, K. Golovin, N. Yan, *Nano Energy* **2023**, *115*, 108752.

[7] D. Nioras, K. Ellinas, E. Gogolides, *ACS Appl. Nano Mater.* **2022**, *5*, 11334-11341.

[8] Hou, Y. Y. Shang, M. Yu, C. Feng, H. Yu, S. Yao, *ACS Nano* **2018**, *12*, 11022-11030.

[9] J. Lin, X. Tan, T. Shi, Z. Tang, G. Liao, *ACS Appl. Mater. Interfaces* **2018**, *10*, 44815-44824.

[10] D. Feldmann, & B. Pinchasik, *J. Colloid. Interf. Sci.* **2023** *644*, 146-156.

[11] K. Park, P. Kim, A. Grinthal, N. He, D. Fox, J. weaver, J. Aizenberg, *Nature* **2016**, *531*, 78-82.

[12] D. Boylan, D. Monga, L. Shan, Z. Guo, X. Dai, *Adv. Funct. Mater.* **2023**, *33*, 2211113.

[13] Z. Guo, L. Zhang, D. Monga, H. Stone, X. Dai, *Cell Reports Physical Science*, **2021**, *2*, 100387.

[14] H. Luo, S. Yin. S. Huang, F. Chen, Q. Tang, X. Li, *Appl. Surf. Sci.* **2019***, 470*, 1139-1147.
